# Ameliorative effects of *Spirulina platensis* niosome and *Echinacea purpura* on cyclophosphamide-induced splenic, cardiac and neurotoxicity via modulating NF-κB pathway and oxidative stress

**DOI:** 10.1038/s41598-026-51198-3

**Published:** 2026-05-11

**Authors:** Shimaa M. Ramadan, Amr Gamal, Nour El-Houda Y. Hassan, Shimaa A. Mahmoud, Walid Hamdy Hassan, Fatma I. Abo El-Ela, Fady Sayed Youssef, Doaa R. I. Abdel-Gawad, Asmaa A. Abo Elqasem

**Affiliations:** 1 Biochemistry Department, Faculty of Science, Beni-Suef University, Beni-Suef, Cairo, Egypt; 2 Department of Pharmaceutics and Industrial Pharmacy, Faculty of Pharmacy, Beni-Suef University, Beni-Suef, Cairo, Egypt; 3 Department of Forensic Medicine and Toxicology, Faculty of Veterinary Medicine, Beni-Suef University, 62511 Beni-Suef, Cairo, Egypt; 4 Biomedical Chemistry, Department of Biotechnology and Life Sciences. Faculty of Postgraduate Studies for Advanced Sciences, Beni-Suef University, Beni-Suef, Cairo, Egypt; 5 Department of Microbiology Mycology and Immunology, Faculty of Veterinary Medicine, Beni-Suef University, 62511 Beni-Suef, Cairo, Egypt; 6 Department of Pharmacology, Faculty of Veterinary Medicine, Beni-Suef University, 62511 Beni-Suef, Cairo, Egypt; 7 Department of Pharmacology, Faculty of Veterinary Medicine, Cairo University, 12211 Cairo, Egypt; 8 Department of Toxicology and Forensic Medicine, Faculty of Veterinary Medicine, Beni-Suef University, 62511 Beni-Suef, Cairo, Egypt; 9 Immunology, Zoology and Entomology Department, Faculty of Science, Al-Azhar University (for Girls), Nasr City, Cairo Egypt

**Keywords:** Cyclophosphamide, *Echinacea purpura*, Haematopiotic, Inflammatory/oxidative makers, *Spirulina platensis*, Biochemistry, Drug discovery, Medical research, Plant sciences

## Abstract

Cyclophosphamide is a widely used chemotherapeutic agent, but its administration is often associated with severe and life-threatening side effects. This study aimed to evaluate the protective efficacy of *Spirulina platensis* noisome and *Echinacea purpurea* against cyclophosphamide-induced splenic, cardiac, and neurotoxic effects. A total of 42 adult male rats were divided equally into seven groups. Group I served as the negative control. Group II was injected with cyclophosphamide. Groups III to VII received various protective oral treatments for 14 days prior to cyclophosphamide: Group III received *Spirulina*, Group IV received *Spirulina* niosome, Group V was treated with *Echinacea*, Group VI received a combination of *Echinacea* and *Spirulina*, and Group VII received a combination of *Echinacea* and *Spirulina* niosome. Hematopoietic parameters, creatine kinase, nuclear factor kappa B (NF-κB), malondialdehyde, nitric oxide, glutathione levels, superoxide dismutase activity, and histopathological examinations were performed. All treated groups showed improved blood profiles, antioxidant status in cardiac and brain tissues, and reduced tissue damage. NF-κB activation was also modulated. These findings suggest that cyclophosphamide induces significant oxidative and inflammatory responses leading to tissue injury. However, administration of *Echinacea* and *Spirulina* offered promising protective effects, proposing a potential novel strategy to counteract cyclophosphamide toxicity.

**Supplementary Information:**

The online version contains supplementary material available at 10.1038/s41598-026-51198-3.

## Introduction

Cancer therapy regimens may involve using chemotherapeutic agents such as anthracyclines and cyclophosphamide that classified as high-risk chemotherapeutic agents have side effects^1^. The chemotherapeutic drugs are cytotoxic acting on variable stages of the cell cycle causing deleterious side effects on both the fast-dividing cells as the gastrointestinal mucosa and bone marrow cells particularly and slow dividing cells as neurons causing neurotoxicity^2^.

Cyclophosphamide (CP) is the most commonly used effective alkylating drug in cancer treatment, but its use is restricted because its toxic side effect causes testicular toxicity^3^. CP is alkylating drugs with antitumor or anti-proliferative effects, they block the cellular replication and repair, in which they cause crosslinks between the double helix strands of DNA at the guanine N-7 position, that ending in the cellular death^4^. CP epitomizes a forceful antineoplastic, immunomodulatory and immunosuppressive agent affecting the cell-mediated and humoral immunity^5^. The CP toxic effects are irreversible and may reach heart failure^6^. The cardiotoxic effect of CP representing lethal CP complications^7^. It is documented that the CP cardiotoxic effects may appear after 48 h from the dose administration and may stay up to 10 days^8^. The neurotoxic effects of CP may be in the form of disturbance of the microglia function^9^, and neuroinflammation^10^ or deleterious effects such as suppression of the production of new cells in the hippocampus^11^ and decreasing the dendritic and spinal complexity Kang et al.^12^.

*Echinacea purpurea* (Ech) has been recently described as a medicinal plant with anti-inflammatory, antioxidant, and immunomodulatory properties. According to Abdel-Aziz et al.^13^*Echinacea* can protect diabetic animals from pulmonary changes. It also appears that *Echinacea* supplementation has a predominantly anti-inflammatory effect on cytokines, including cytokines that play a crucial role in the progression of severe COVID-19. In their study Kolev et al.^14^ determined that commercially available *Echinacea* extract, in its licensed dosage (Echinaforce extract), was a safe, easy-to-use, and widely available antiviral that reduced virus load and prevented respiratory tract infections, including SARS-CoV2. Wahba^15^ denoted that diet supplementation with *Echinacea* exhibits good immunostimulant, hypoglycemic and antioxidant activities. In 1984, Stimpel et al.^16^ provided evidence that *Echinacea* polysaccharides activated macrophages to eliminate tumor cells while having no impact on T or B lymphocytes. Khalaf et al.^17^ reported that *Echinacea* pretreatment provided a protective effect against cisplatin-induced immunotoxicity by detecting mild or negative caspase-3 immunoreactivity. The Abdelmotaleb et al.^18^ study’s findings demonstrate the *Echinacea* ethyl acetate fraction’s potential for use in the creation of novel antibiotics that combat multidrug-resistant bacteria.

Historically, humans have consumed *Spirulina plantensis* (SP) as a health drink and tablet since it is rich in protein and vitamins^19^. According to a number of studies, *Spirulina* species possess a variety of biological properties, including antioxidants, anti-inflammatory effects, immunomodulation^20,21^, antitumor effects, neuro-protective effects^22^, radioprotective effects^23^ and metaloprotective properties^24^. Previous studies have identified the main protein in SP, phycocyanin-C, as responsible for these effects. Previous study showed that the antioxidant potential of the phycocyanin present in cyanobacterium SP indicated that the amount administered to the rats was sufficient to reduce and also prevent the oxidative damage caused by monosodium glutamate in vivo^25^.

In another study, researchers explored the use of *Spirulina plantensis* extract as a natural alternative to synthetic plant growth regulators for the in vitro propagation of Capparis cartilaginea, a medicinal plant known for its propagation challenges. The findings demonstrated that supplementing the culture medium with *Spirulina* extract significantly enhanced shoot length and leaf number. Additionally, the extract increased the phenolic content in the plant tissue. High performance liquid chromatography analysis revealed the presence of phytohormones such as gibberellins, kinetin and adenine indicating its potential as a cost-effective and natural growth promotor and plant tissue culture applications^26^. The El-Sayed et al.^27^ study examined the antibacterial activity of phenolic acid extract from *Spirulina* against pathogenic bacteria that cause sepsis in newborns.

Numerous research has examined vesicular systems at the nanoscale, including liposomes and niosome, which represent a more recent class of vesicular nanocarriers. In the self-assembled vesicle, which is made up of non-ionic surfactants combined with cholesterol or other amphiphilic molecules, the niosome serve as a multilamellar carrier for lipophilic and hydrophilic bioactive compounds. There are numerous technological uses for these non-ionic surfactant vesicles, also referred to as niosome. Niosome are thought to be more chemically and physically stable than liposomes. The niosome preparation techniques are more cost-effective^28^.

Despite the reported antioxidant and immunomodulatory properties of *Spirulina platensis* and *Echinacea purpura*, limited studies have evaluated their combined protective potential, particularly in nano formulations against the CP-induced multi-organ toxicity. Furthermore, the role of *Spirulina* niosome in enhancing bioavailability and modulating inflammatory signaling pathways such as NF-κB in splenic, cardiac and neural tissues remains insufficiently explored. Therefore, the present study aimed to investigate the protective effects of *platensis* (in its free or loaded niosome formulations) and *Echinacea purpura*, individually and in combination against CP-induced toxicity. We hypothesized that these treatments would mitigate oxidative stress and inflammatory responses through modulation of NF-κB therefore reducing tissue damage in spleen, heart and brain.

## Materials and methods

### Cyclophosphamide (CP) and *Echinacea purpura* (Ech) extract

Cyclophosphamide (CP) was obtained from Sigma-Aldrich Scientific International, Inc., “Sigma-Aldrich Co. (St Louis, MO, USA)”. provided *Echinacea purpura* extract with zero fillers, preservatives, sugars, sweeteners, and chemicals from Multi-pharma Company in Cairo.

### *Spirulina platensis* (SP) preparations

As soon as the green microalgae *Spirulina platensis* were obtained, the algae were collected and kept in an alkaline medium pH, which allowed the algae to grow in the summer. Once the *Spirulina* has grown, the water is suctioned from the ponds and the water is collected using silk cloths with micro-holes that filter the algae. In addition to passing through smaller holes than the afore mentioned, it is also exposed to a hot air current after it has been passed through this process more than once. The air is suctioned onto this silk cloth to dry the *Spirulina*. In the next step, it is collected, ground, and finally sterilized with ultraviolet radiation and provided as a pure green powder.

### Loading of *Spirulina Platensis* with niosome (SPN) and Characterization

#### Loading

We designed SP-loaded niosome using thin film hydration^29^. A 1:2:0.1 mixtures of tween 60, cholesterol, and dihexadecyl phosphate (DDP) were dissolved in chloroform/methanol. Then, we evaporated the organic solution under vacuum at 60 °C and hydrated the thin film with an aqueous solution of SP (10 mg) for 2 h. For further investigation, we refrigerated the prepared SPN formulation.

#### Characterization

##### Transmission electrons microscope (TEM)

An electron microscope (Carl Zeiss, Germany) was used to examine the morphology of SPN vesicles. TEM (70 kV voltage) was used to capture the image of 20 µl of SPN formulation deposited on a carbon-coated copper grid, dyed with phosphotungstic dye, and allowed to dry.

##### Analysis of particle size, polydispersity indexes and zeta potential

Nowroozi et al.^30^ discuss how particle size and poly dispersity index (PDI) affect dispersion, homogeneity, and distribution of particles, and therefore their ability to be targeted. A zeta potential assessment of SPN formulations allowed them to be evaluated electrostatically, for surface properties, and for stability. Dynamic light scattering (DLS, Germany) was used to evaluate particle size, poly dispersity index (PDI), and zeta potential of three duplicates of each *Spirulina* niosome (SPN) formulation sample (1 ml)^31^.

### Experimental Animals

Laboratory animals (healthy 42 male albino rats with weights ranging from 150 to 250) were obtained from Beni-Suef University’s Pharmaceutical Department, Laboratory Animal Unit. Beni-Suef University’s Faculty of Veterinary Medicine’s institutional animal care and use committee oversees all animal handling, weighing, dosing, and slaughtering during the experimental period. Clean fresh water, standard diets, and a 12-hour dark-light cycle were used throughout the experiment.

### Experimental design

**Fig. 1:**
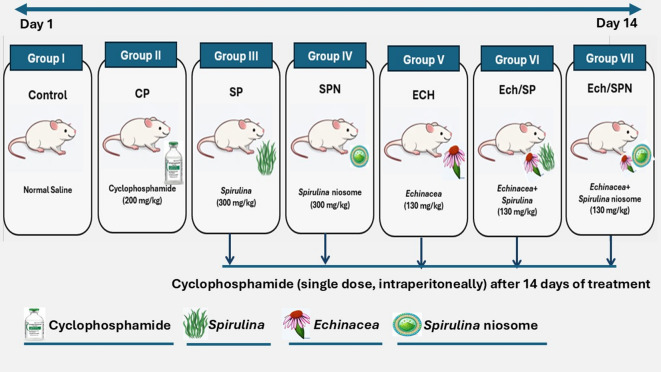
Experimental design showing treatment groups and dosing schedule.

Healthy animals were distributed into seven groups equally. Group I represent negative control group (receive 1.5 ml/kg of saline oral gavage). Group II as a positive control group (receiving the toxic CP at a dose (200 mg/kg)^32^. The rest groups receiving the protective natural products orally for about 14 days daily, in which Group III received SP and Group IV administered SPN at a dose (300 mg/kg)^33^, while Group V given ECH, Group VI given Ech/SP, and Group VII received Ech/SPN at a dose (130 mg/kg)^34^. Following 14 day, Group II and the groups received the protective therapy were intraperitoneal injected CP as a single dose (Fig. 1). The animals’ body weight was recorded on the 7th and 15th days of the experiment. At the end of the experiment all rats were deeply anesthetized before euthanasia. Euthanasia was performed by cervical dislocation, and the death was confirmed by lack of reflexes.

### Samples collection

Twenty-four hr. following CP injection, rats were anesthetized with a 1:1 xylazine/ketamine intraperitoneal (0.1 ml/100 g). Blood samples were collected in heparinized microhematocrit tubes to obtain plasma. In order to obtain serum samples, blood samples were centrifuged for 15 min at 3000 °C and stored at −20 °C. Heart, spleen, and brain samples were collected and washed with 0.9% normal saline. For the removal of red blood cells, 50 mg/l of ice-cold Na2HPO4/NaH2PO4 (pH 7.4) was added. For further analysis of antioxidant biomarkers and lipid peroxidation, tissue samples were ground in ice-cold buffer at 1 g per 5–10 mL after centrifugation for 30 min at 3000 rpm for 30 min. Supernatants were stored at − 80 °C. Other parts of the tissues were kept for the histopathological examination.

### Haematological investigation “complete blood picture” (CBC) assays

For the haematological assays, blood samples were coagulated for1 h at room temperature before centrifugation in a refrigerated centrifuge (Sigma) for 10 min at 4000 rpm. An automated hematology analyzer (Sysmax-18i, Japan) was used to perform the CBC including red blood cell (RBC), white blood cell (WBC), eosinophils, lymphocytes, monocytes, neutrophils, platelets, hemoglobin (Hb), mean corpuscular volume (MCV), mean corpuscular hemoglobin (MCH), hematocrit (HCT), mean cell hemoglobin concentration (MCHC). RBCs were counted by hemocytometer chamber as described by Sahastrabuddhe^35^. Both Differential leucocytic count was determined and WBCs count was done according to Jain^36^, by using the trucks solution as diluent for WBCs. Thin homogenous blood films were prepared on clean dry slides and stained with Leishman’s stain. The percentage of each type of white cells was noted after counting 100 white cells per slide. The packed cell volume (PCV) was verified according to the method clarified by Bull^37^. The hemoglobin cyanide method was used to determine the Hb concentration^38^.

### Assessment of cardiac function, oxidative stress, and inflammatory markers

#### Cardiac function indicator (CK-MB) enzyme

A quantitative assessment of creatinine kinase enzyme (CK-MB) was performed. The antibodies inhibit subunit M of CK-MB and NADPH is measured photometrically when non inhibited CK-MB undergoes a series of reactions. The CK-MB unit per liter can be calculated using Würzburg et al.^39^ calculation.

#### Evaluation of tissue oxidant and antioxidant enzyme activities

Studies have been conducted by Aebi^40^, Paglia et al.^41^ and Nishikimi et al.^42^ to measure the activities of SOD and GSH in cardiac, and brain organs. Beutler et al.^43^ measured tissue GSH levels. Green et al.^44^ and Kir et al.^45^ examined tissue NO and MDA.

#### Assessment of nuclear factor kappa (NF-κB) *via* enzyme-linked immunosorbent assay (ELISA)

ELISA was used to determine the levels of NF-κB in rat sera, performed according to the method of Brouckaert et al.^46^ rat NF-κB ELISA kit catalogue number is ER1186. In spectrophotometry, the color change is measured at 450 nm±10 nm.

### Histopathological investigation

Spleen, heart, and brain specimens were collected on day 15 post-surgery and conserved in a solution of 10% neutral buffered formalin. Subsequently, these samples underwent standard procedures for processing, for staining and examination, a light microscope was used. Based on the methodology, histological lesion scores were determined as described by Hosseini et al.^47^. Additionally, further tissue slides were subjected to staining using Masson’s trichrome stain (MTC) to assess the deposition of collagen fibers in the dermal layer. The quantification and statistical analysis of collagen fibers were conducted based on their area %. And on their maturity because the immature collagen tissue shows more cellularity while mature one has low cellular content.

### Statistical analysis

Statistical analyses were carried out using SPSS (version 20.0, IBM SPSS Statistic, Armonk, NY, USA) software. Data were represented as mean ± standard errors of the means (SEMs). One-way analysis of variance (ANOVA) was used to determine differences among groups. Normality was assessed using the Shapiro–Wilk test, and homogeneity of variance was assessed using Levene’s test. When significant differences were detected, pairwise group comparisons were performed using the Turkey’s post hoc test. A p value < 0.05 was considered statistically significant.

## Results

### Characterization of *Spirulina Platensis *Niosome (SPN)

#### Transmission Electron Microscope (TEM) analysis

**Fig. 2:**
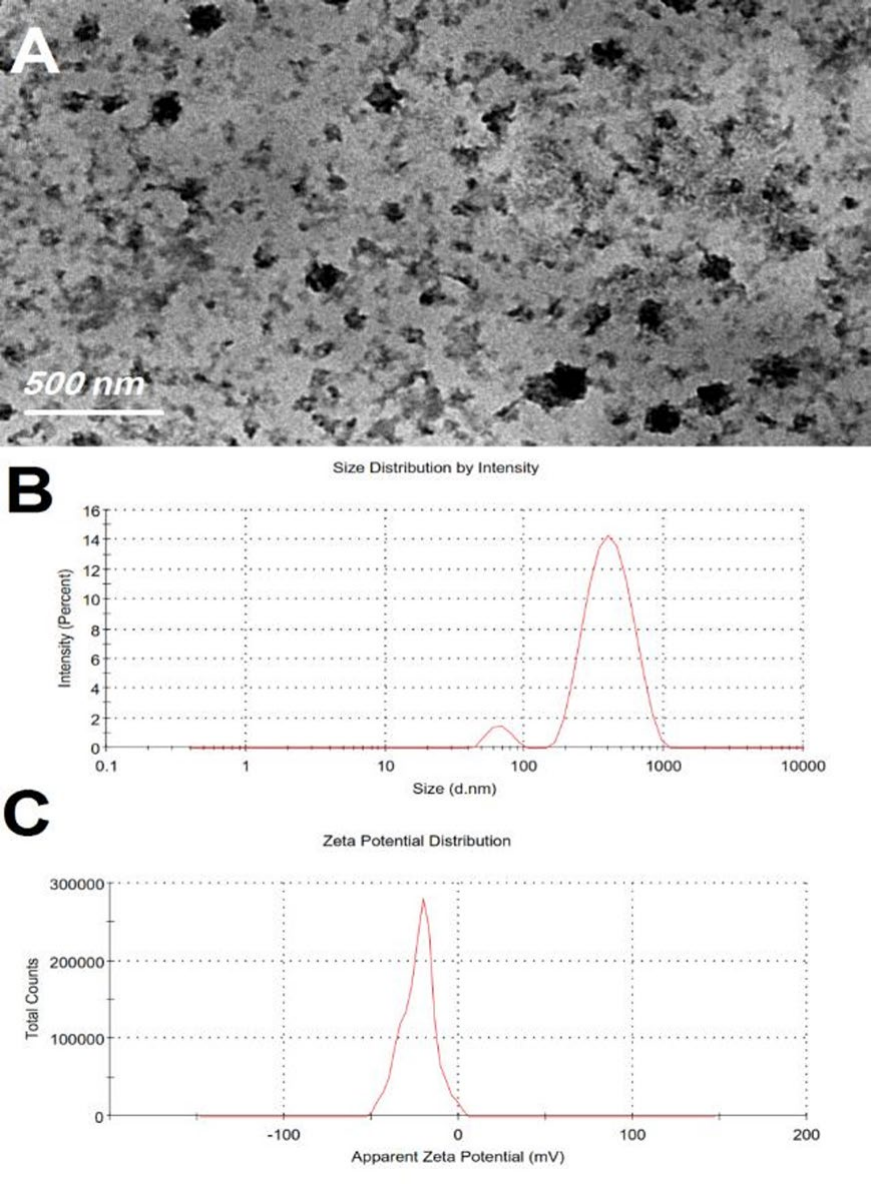
Transmission electron microscopy showing obvious SPN formulations without aggregation and successful loading of SP into niosomal Nano carriers **(A)**; Size distribution by intensity of SPN formulations **(B)**; Zeta potential spectrum of the SPN formula **(C)**.

As determined by the Malvern Zetasizer, TEM images of the SPN formulation showed nano-sized spherical particles. The TEM analysis of SPN formulations also showed no aggregation. As a result, it was observed that Nano carriers loaded with SPN formulations effectively as shown in Fig. 2A.

#### Analysis of particle size, polydispersity indexes and zeta potential

SPN uses a particle size distribution with a uniform size and a low PDI of 0.405, indicating a homogeneous noise distribution without aggregation (Fig. 2B). The SPN formulation’s zeta potential was tested and determined to be −23.2 ± 0.36 (Fig. 2C), highlighting the great stability and purity of prepared SPN.

### Body weight changes

**Fig. 3:**
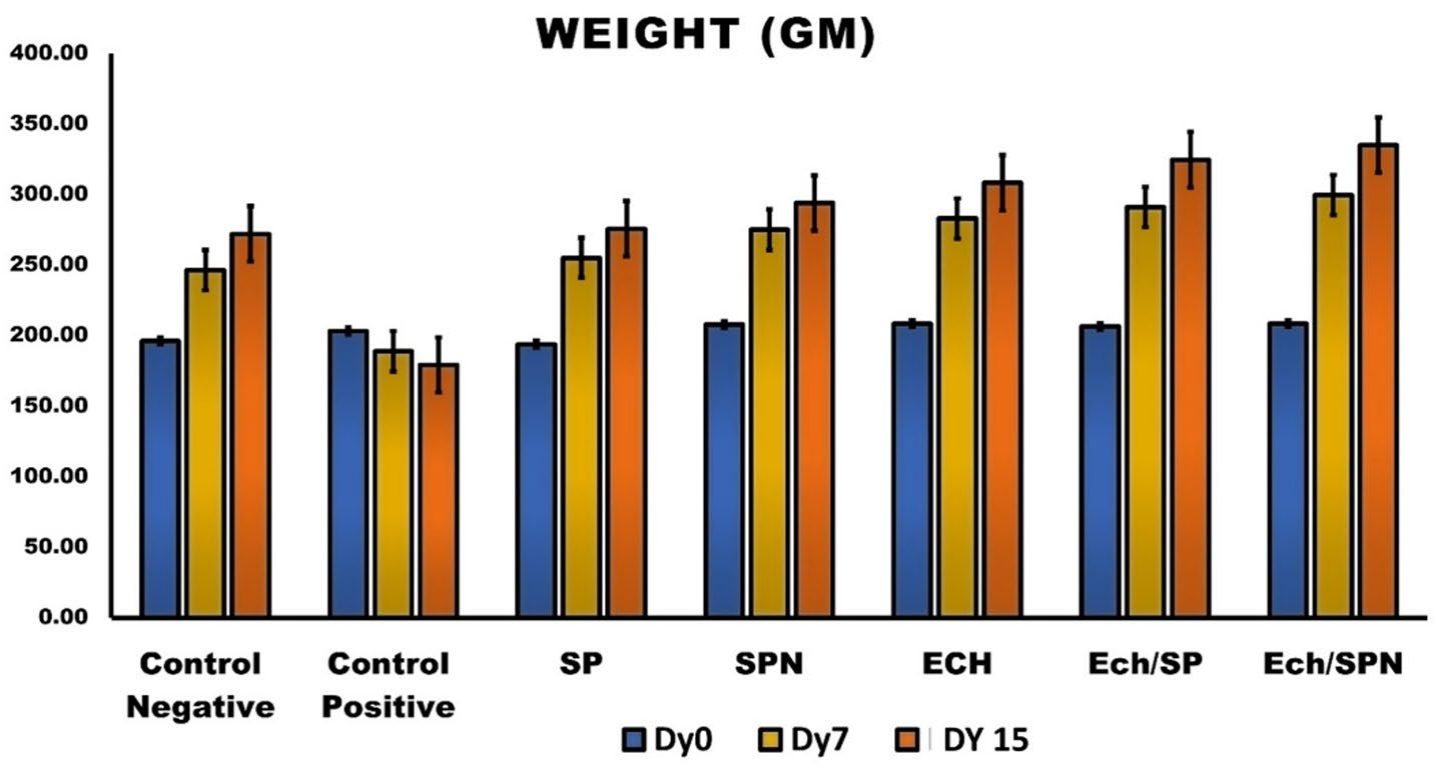
The effect of oral administration of SP, SPN, ECH, Ech/SP and Ech/SPN on body weight of CP—intoxicated rats. Data are presented as mean ± SEM.

The substances SP, SPN, ECH, Ech/SP, and Ech/SPN on body weight on the 7th and 15th days following treatment. On the 7th and 15th day after administering SP, the groups treated with SPN showed insignificant increases in body weight compared to the control negative groups (*p* > 0.05), but significant increases compared to the CP-intoxicated animals (*P* < 0.05). The groups treated with ECH, Ech/SP, and Ech/SPN showed a significant increase in body weight compared to the control negative group and the CP-intoxicated (Fig. 3).

### Haematological investigation (Complete Blood Picture) (CBC)

When compared to all treated and control negative groups, rats intoxicated with CP showed non-significant elevation of Hb levels. Comparatively to all treated groups and control groups, the RBCs count was significantly decreased in the CP intoxicated group. Compared with the CP-intoxicated group, the HCT % was significantly lower in the Ech/SP and Ech/SPN treated groups. In contrast to CP intoxicated group, SPN, ECH, Ech/SP, and Ech/SPN treated groups showed significantly lower MCV levels.

There was a significant increase in the MCH value in rats intoxicated with CP as compared to rats supplemented with SPN, ECH, Ech/SP, and Ech/SPN. There was a significant decrease in MCHC in the Ech/SP treated group, and also in the Ech/SPN treated group, as compared to the CP-intoxicated group. Compared to other treated groups, RDW values were significantly reduced in the CP-intoxicated group. Both CP-intoxicated and SP-treated groups had significantly elevated MCV/RBCs (Mentzer Index) compared to other treated groups. Compared with other treated groups, SP and Ech/SP treated groups had significantly lower platelet counts. Compared to CP-intoxicated group, PCT% value was significantly lower in SPN, Ech/SP, and Ech/SPN. A significant increase in MPV values was observed in the CP-intoxicated group as compared to other groups and the control non-treated group, *p* < 0.05.

**Fig. 4:**
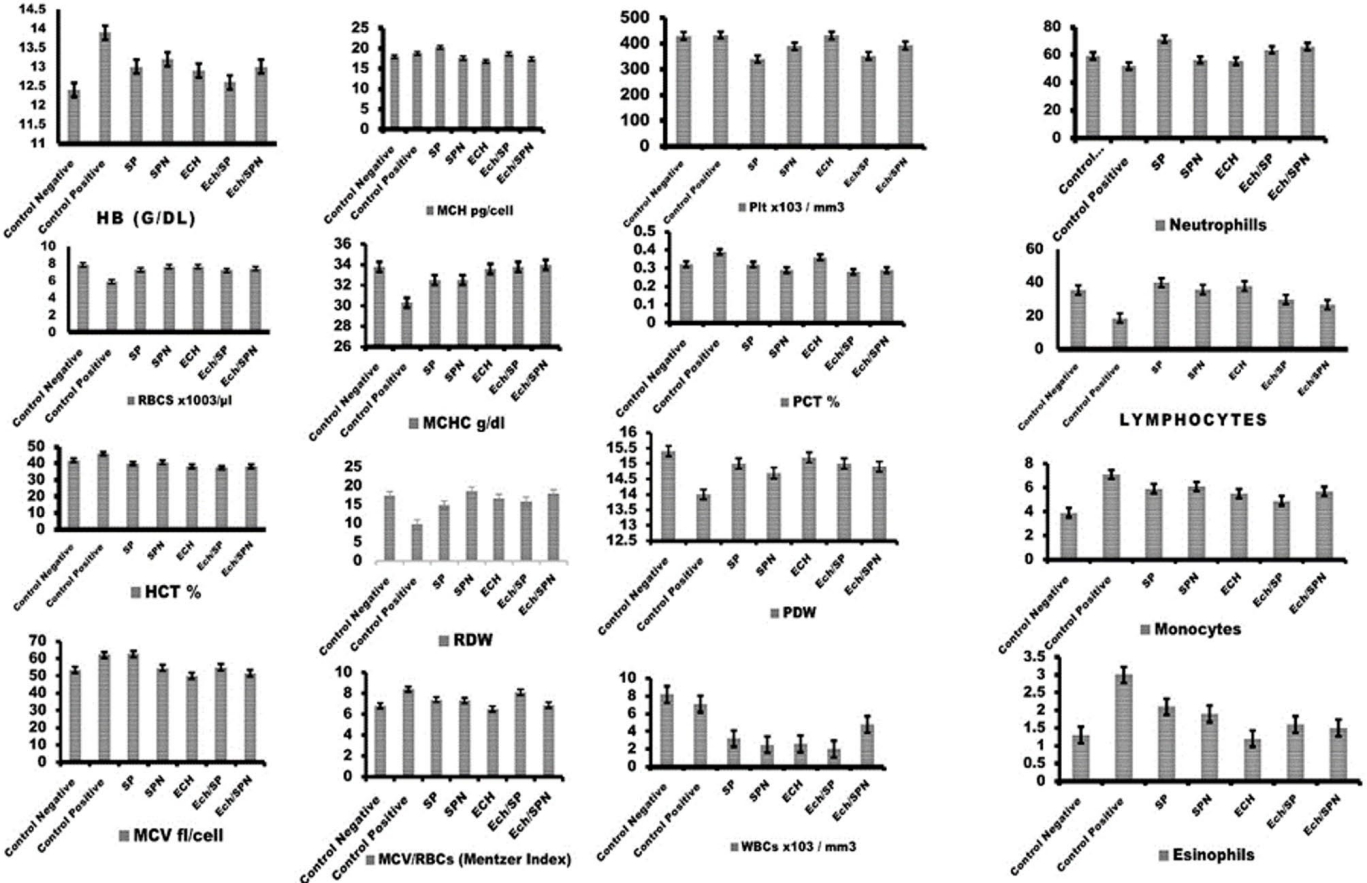
The effect of oral administration of SP, SPN, ECH, ECH/SP and ECH/SPN on complete blood picture (CBC) of CP-intoxicated rats. Data are presented from (A to Q) as mean ± SEM.

CP-intoxicated group PDW values showed no significant difference from other groups or the non-treated control group. According to Fig. 4, the WBC count was significantly reduced in the SPN, ECH, Ech/SP, and Ech/SPN groups when compared to the CP-intoxicated group. In comparison with the CP-intoxicated group, the neutrophil count was significantly higher in the SPN, ECH, Ech/SP, and Ech/SPN treated groups. In comparison to the CP-intoxicated group, the lymphocyte count was significantly higher in SPN, ECH, Ech/SP, and Ech/SPN. As compared to control negative groups, all treated groups showed significant elevations in monocyte counts. When compared to all treated groups and normal control groups, the eosinophilic count increased significantly (Fig. 4).

### Cardiac function indicator; Creatine Kinase MB (CK-MB levels)

**Fig. 5:**
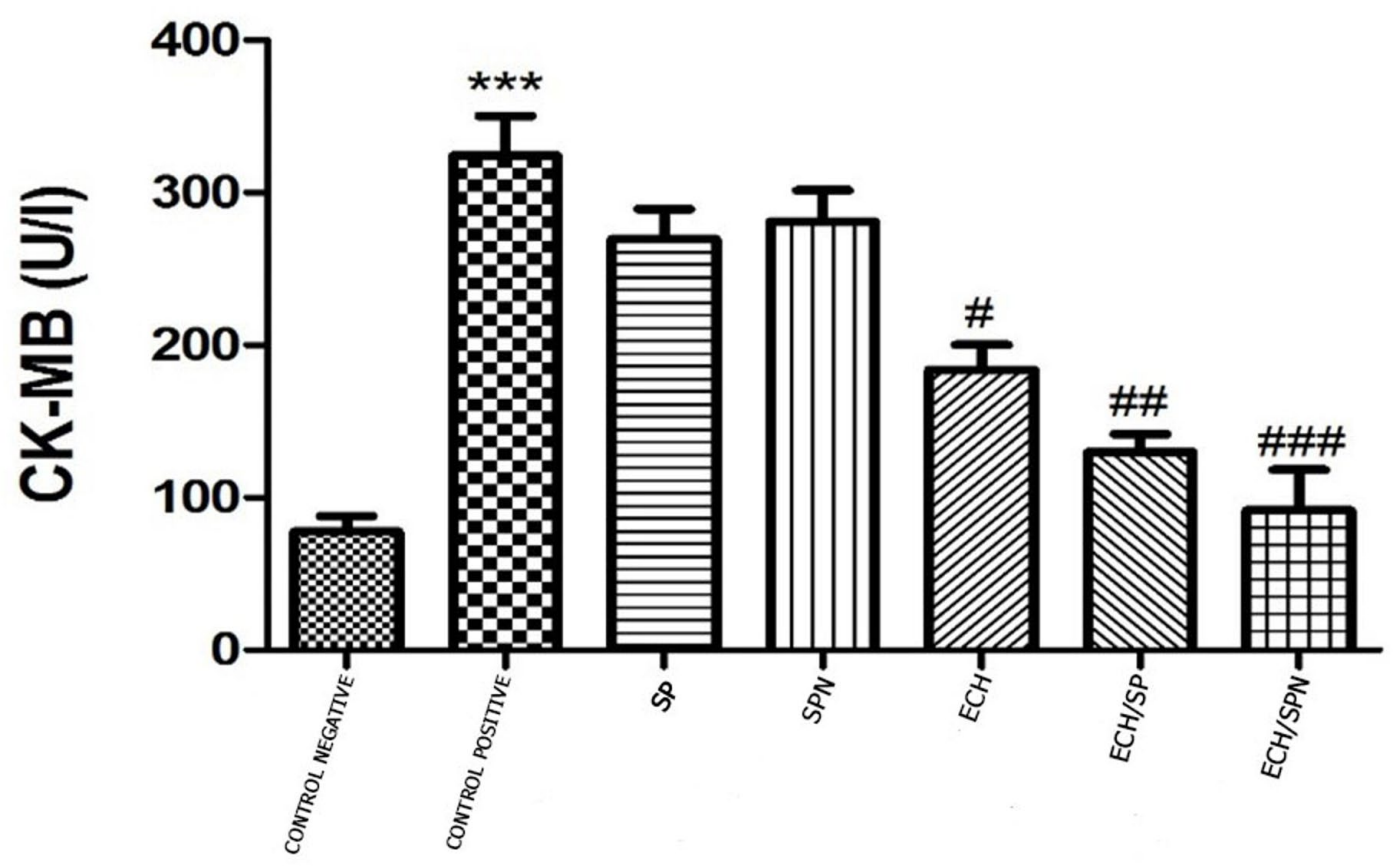
Effect of SP, SPN, ECH, ECH/SP and ECH/SPN on serum CK-MB level in CP-induced rats. Data are expressed as mean ± SEM.***p < 0.001 versus Control, and ^#^*p* < 0.05, ^##^*p* < 0.01 and ^###^*P* < 0.001 versus CP. CK-MB; Creatine kinase-MB, SP: Spirulina, SPN: Spirulina noisome, ECH: Echinacea, Ech/SP: Echinacea + Spirulina, Ech/SPN: Echinacea + Spirulina noisome.

The potential of SP, SPN, ECH, Ech/SP, and Ech/SPN to improve CP-induced cardiotoxicity was evaluated by measuring CK-MB levels (Fig. 5). The administration of CP resulted in a substantial increase in serum CK-MB levels compared to the control rats, with a statistical significance of *P* < 0.001. Administering either SP or SPN orally resulted in a non-significant decrease in serum CK-MB levels compared to rats intoxicated with CP. Nevertheless, the administration of ECH, ECH/SP, and ECH/SPN resulted in a substantial reduction in serum CK-MB levels compared to rats intoxicated with CP, with p-values of less than 0.05, 0.01, and 0.001 respectively.

### Oxidant and antioxidant analysis

#### Cardiac tissue

**Fig. 6:**
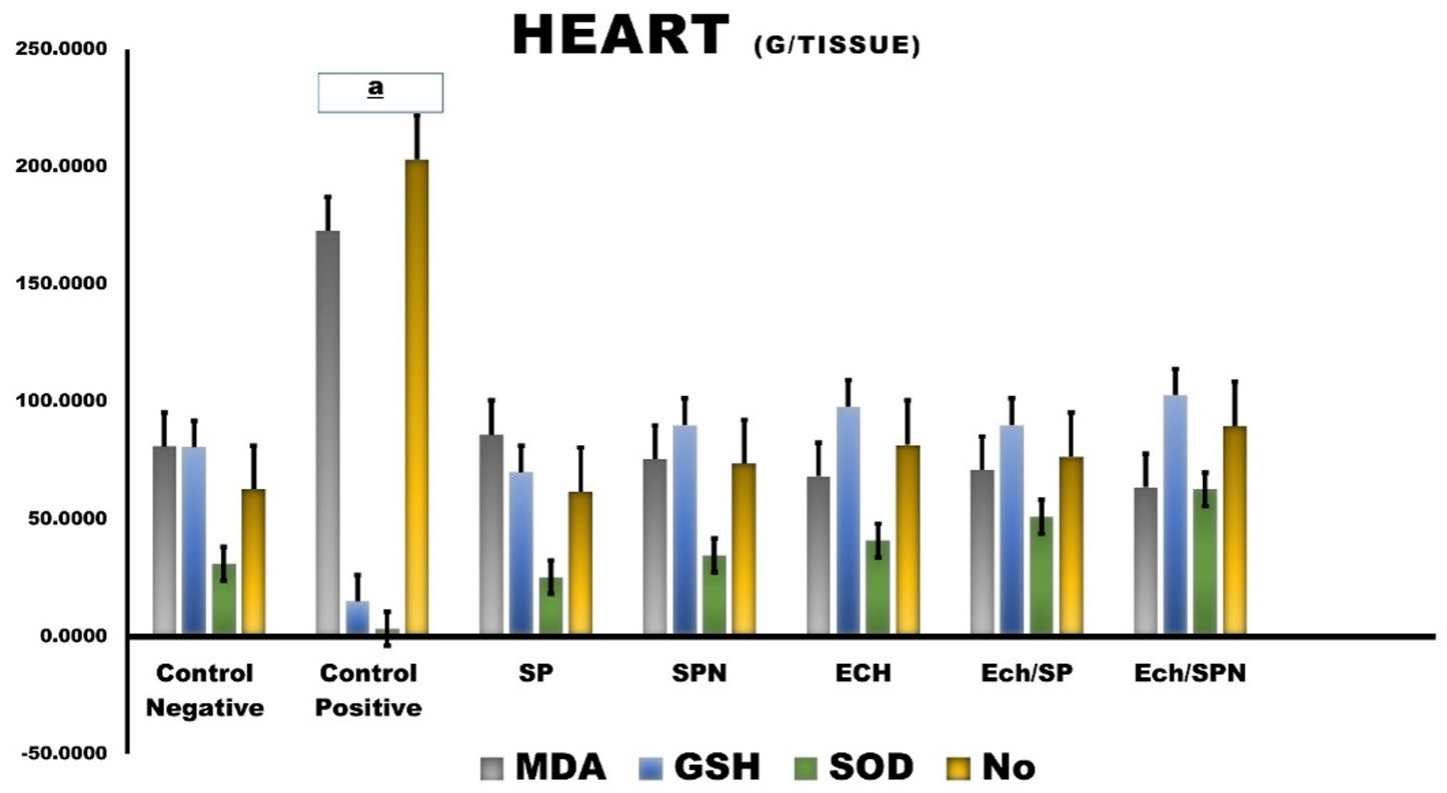
The antioxidant effects of Effect of SP, SPN, ECH, Ech/SP and Ech/SPN against CP–induced hepatotoxicity on MDA, malondialdehyde; NO, nitric oxide; GSH, reduced glutathione; SOD, superoxide dismutase; in cardiac tissue. Data are presented as mean ± SEM. The statistical significance of ^a^*p*<0.05 was seen in comparison to the negative control.

Malondialdehyde (MDA) and Nitric oxide (NO) concentrations in cardiac tissues are significantly higher in CP-treated rats, whereas Glutathione (GSH) and Superoxide Dismutase (SOD) concentrations are lower. When compared to CP-administered rats, SP and SPN significantly reduced MDA and NO activity in cardiac tissue while increasing GSH and SOD levels **(***P* < 0.05). Also, ECH, Ech/SP, and Ech/SPN exhibit significant increased cardiac tissue levels of GSH and SOD (*P* < 0.05). As a result, ECH/SPN outperformed the other treatments (Fig. 6).

#### Brain tissue

**Fig. 7:**
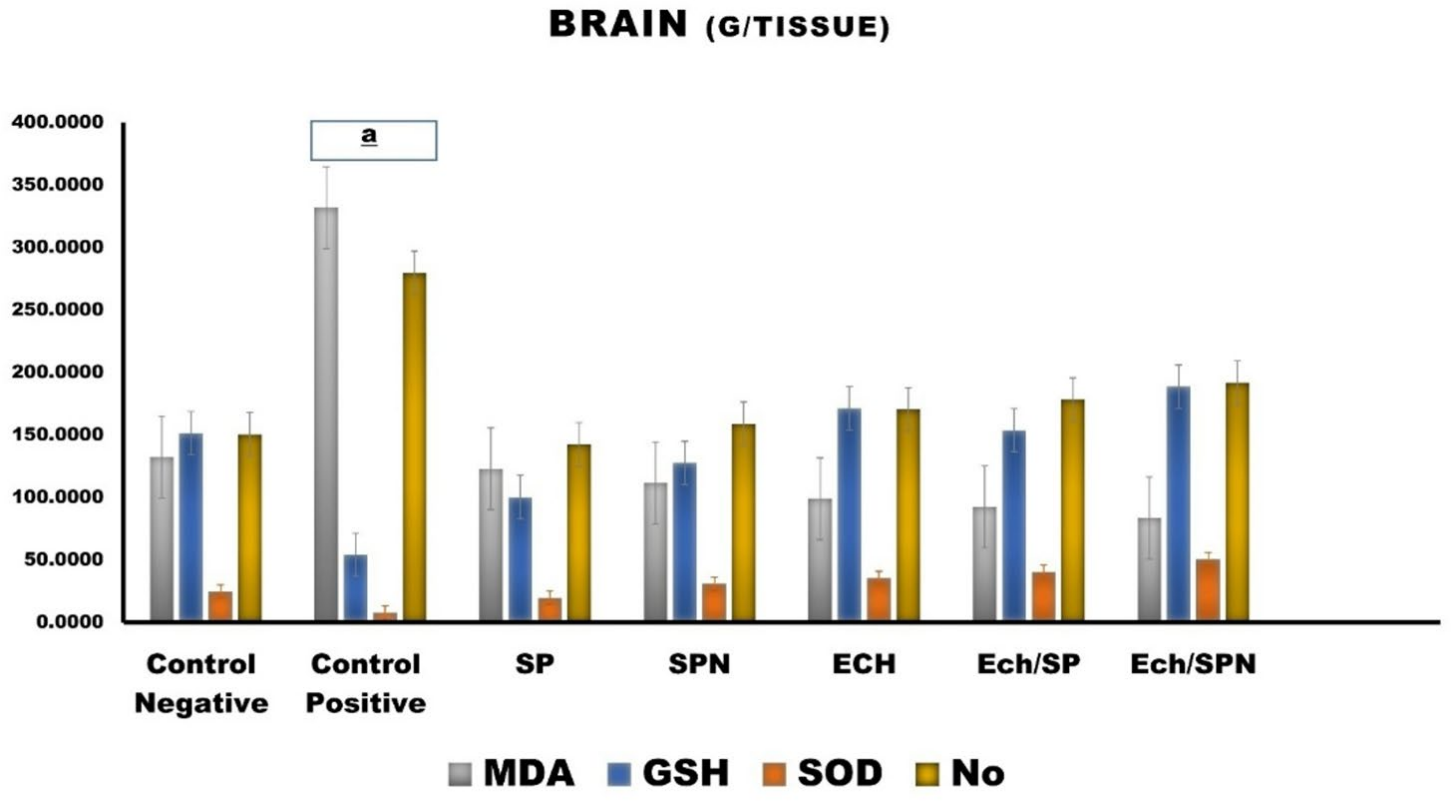
The antioxidant effects of Effect of SP, SPN, ECH, ECH/SP and ECH/SPN against CP–induced hepatotoxicity on MDA, malondialdehyde; NO, nitric oxide; GSH, reduced glutathione; SOD, superoxide dismutase in cerebral tissue Data are presented as mean ± SEM. *The statistical significance of* ^*a*^*p*<0.05 was seen in comparison to the negative control.

The study focuses on the oxidation of lipids in the brain and the level of antioxidants. Figure 7 displays the outcomes of rats that were administered Ech/SP and Ech/SPN. Exhibited statistically significant differences (*p* < 0.05) compared to the positive control group in all measures of oxidative stress and antioxidant activity, including substantial differences (*p* < 0.05). The intervention improved all changes caused by CP, restoring the brain tissue’s concentrations of MDA, NO, and GSH to normal ranges. CP intoxication resulted in elevated levels of MDA and NO in brain tissue (*P* < 0.05), while GSH levels were decreased (Fig. 7).

### The level of nuclear factor kappa (NF-κB)

**Fig. 8:**
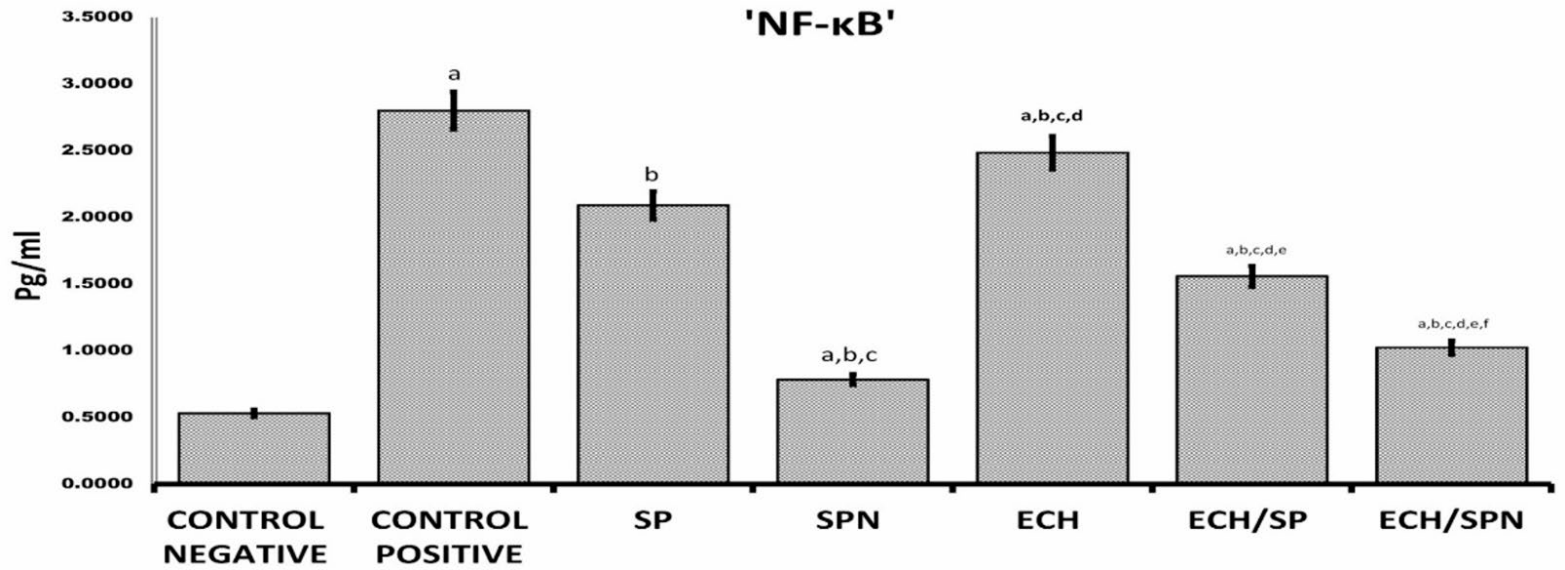
Effect of SP, SPN, ECH, ECH/SP and ECH/SPN on NF-kB serum level of CP-induced rats. Each value represented the mean ± standard error (SE). The statistical significance of ^a^*p*<0.05 was seen in comparison to the negative control. Similarly, ^b^*p*<0.05 was found in relation to positive control. Additionally, ^c^*p*<0.05 was observed compared to SP (oral), and ^d^*p*<0.05 was found relative to SPN, ^e^ *p* < 0.05 was observed compared to ECH/SP and ^f^ *p* < 0.05 was found relative to ECH/SPN.

The intoxicated rats with CP exhibited a highly significant elevation in NF-kB, when compared to both the negative control group and other groups. The SPN and Ech/SPN groups exhibited marginally elevated level of NF-κB mediator compared to the negative control and other treated groups. The ECH, SP, and Ech/SP groups exhibited a notable elevation in serum NF-κB levels, as depicted in Fig. 8. Prior to ANOVA, data were tested for normality using the Shapiro–Wilk test and for homogeneity of variances using Levene’s test. No significant violations of these assumptions were detected (*p* > 0.05) and therefore all variables met the criteria for parametric analysis.

### Histopathological investigations

**Fig. 9:**
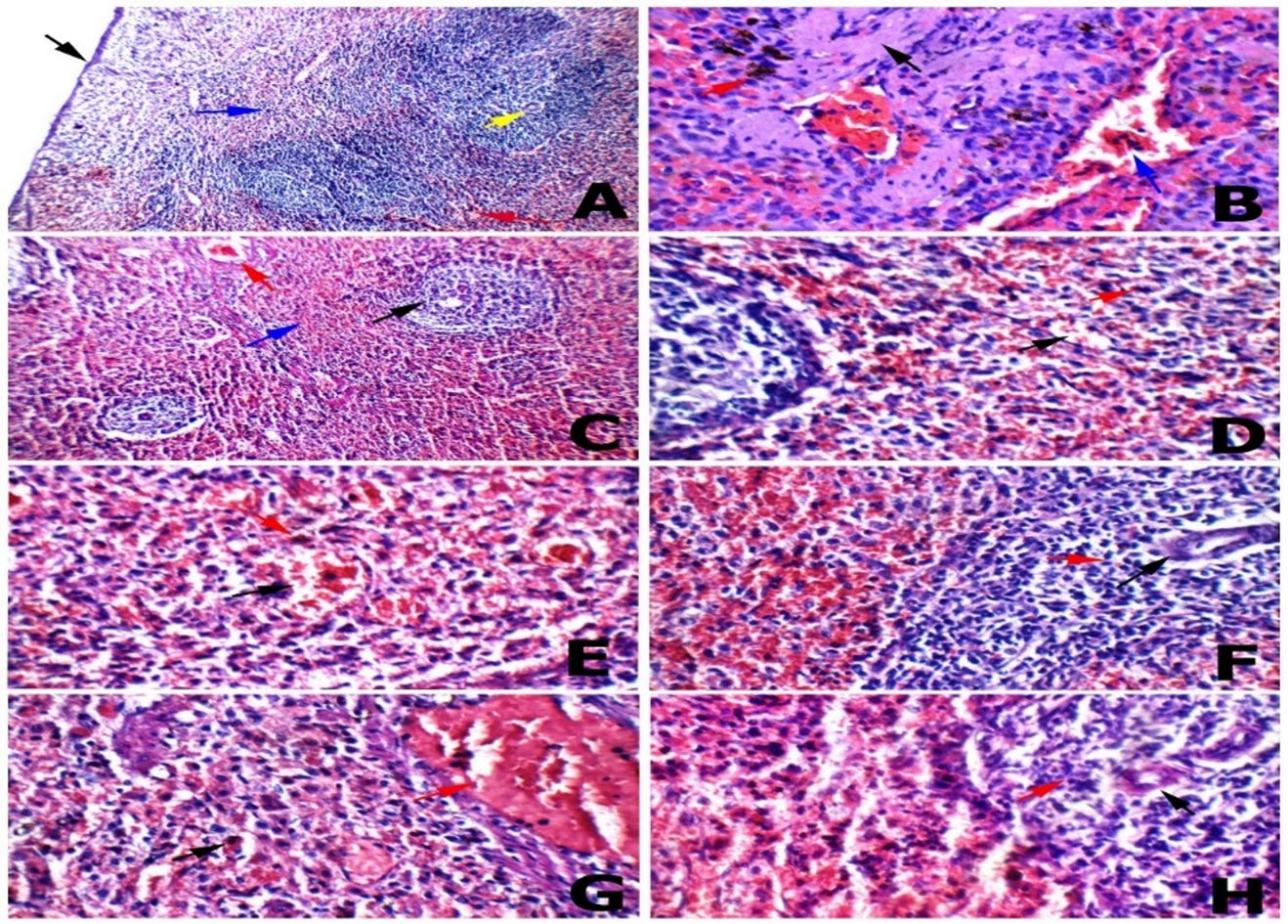
Histopathological investigation of spleen tissue **(A) Negative control**: spleen showing average capsule (black arrow), average lymphoid follicles (white bulb) with central arterioles (yellow arrow), average blood sinusoids (red bulb) (blue arrow), and average blood vessels (red arrow) (H&E x 200). **(B) Positive control**: another view showing atrophied lymphoid follicles with marked hyalinosis (black arrow), and markedly dilated congested blood vessels (blue arrow) with excess ciderophages (red arrow) (H&E x 400). **(C) SP 1**: spleen showing small-sized lymphoid follicles with central arterioles (black arrow), expanded (red bulb) (blue arrow), and mildly congested blood vessels (red arrow) (H&E x 200). **(D) SPN 1**: another view showing average blood sinusoids (red bulb) (black arrow), with scattered ciderophages (red arrow) (H&E x 400). **(E) ECH 1**: another view showing average blood sinusoids (red bulb) (black arrow), with scattered ciderophages (red arrow) (H&E x 400). **(F) ECH + SP**: high power view showing small-sized lymphoid follicles with central arterioles (black arrow), and average lymphocytes in peri-arteriolar area (red arrow). **(G) ECH + SPN**: another view showing markedly expanded red bulb with scattered ciderophages (black arrow), and markedly congested blood vessels (red arrow) (H&E x 400). **(H) ECH + SPN**: high power view showing atrophied lymphoid follicles with central arterioles (black arrow), and average lymphocytes in peri-arteriolar area (red arrow).

**Fig. 10:**
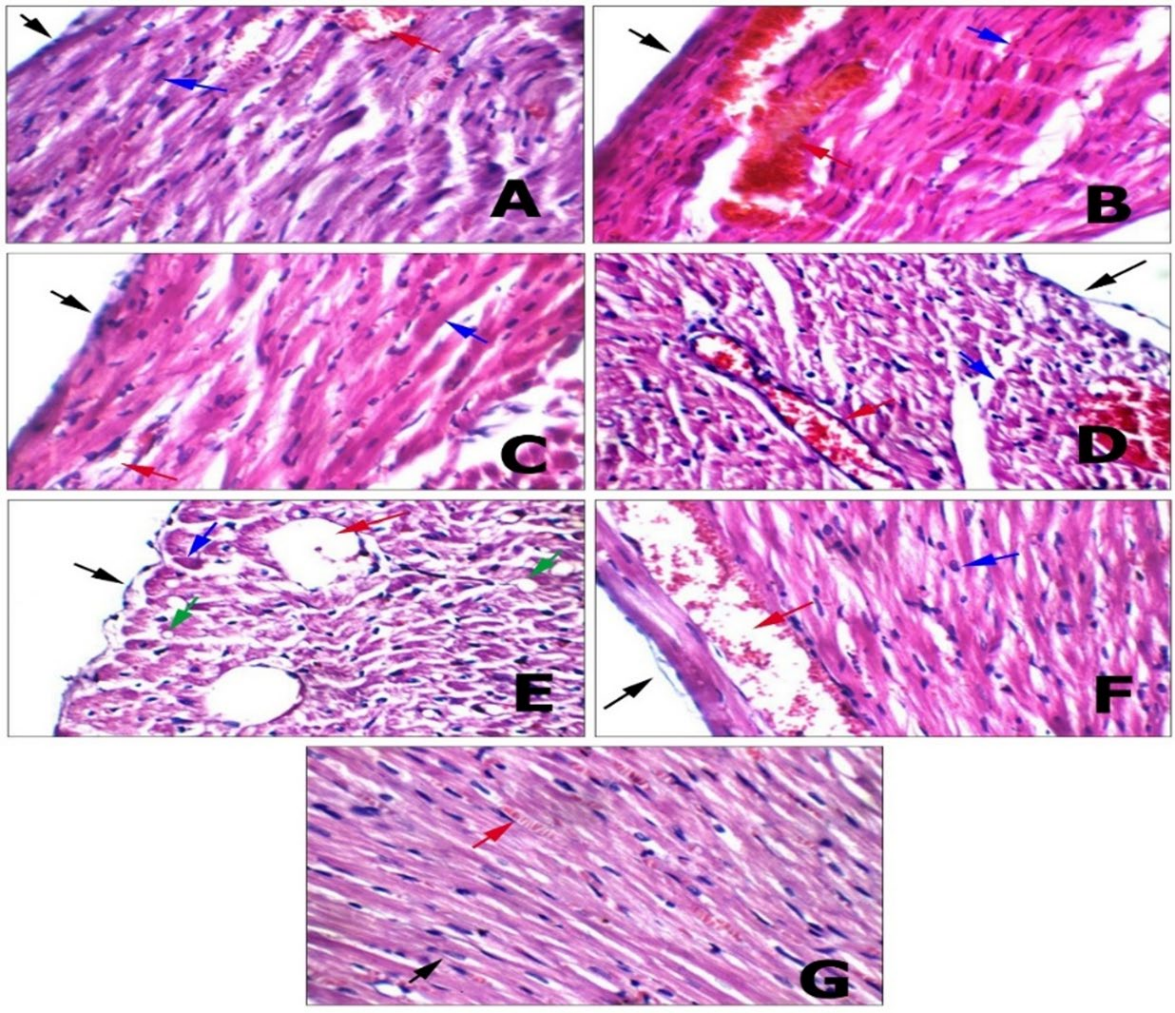
Histopathological investigation of cardiac tissue. **(A) Negative control**: high power view showing intact pericardium (black arrow), viable cardiac muscle fibers with central oval\elongated nuclei (blue arrow), and average myocardial blood vessels (red arrow) (H&E X 400). **(B) Positive control**: cardiac wall showing intact pericardium (black arrow), markedly apoptotic cardiac muscle fibers (blue arrow), and markedly congested sub-pericardial blood vessels (red arrow) (H&E X 400). **(C) SP 1**: high power view showing intact pericardium (black arrow), viable cardiac muscle fibers with central oval\elongated nuclei (blue arrow), and average blood vessels (red arrow) (H&E X 400). **(D) SPN 1**: high power view showing intact pericardium (black arrow), scattered apoptotic cardiac muscle fibers (blue arrow), and mildly congested blood vessels (red arrow) (H&E X 400). **(E) ECH 1**: high power view showing intact pericardium (black arrow), scattered apoptotic cardiac muscle fibers (blue arrow) and others with small cytoplasmic vacuoles (green arrow), and mildly dilated blood vessels (red arrow) (H&E X 400). **(F) ECH + SP**: high power view showing intact pericardium (black arrow), average cardiac muscle fibers with average nuclei (blue arrow), and mildly dilated blood vessels (red arrow) (H&E X 400). **(G) ECH + SPN**: another view showing average cardiac muscle fibers with average nuclei (black arrow), and average intervening capillaries (red arrow) (H&E X 400).

**Fig. 11:**
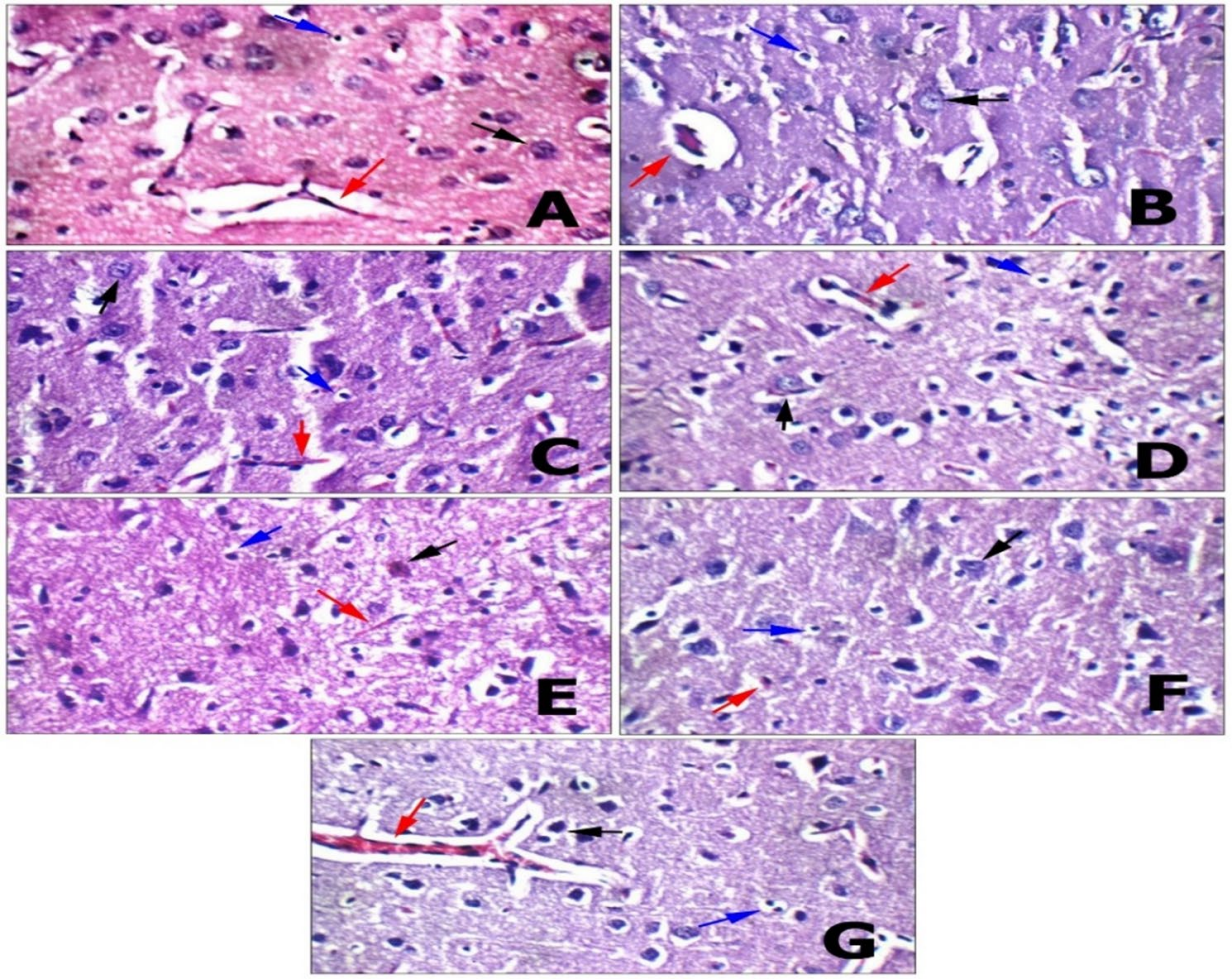
Histopathological investigation of Brain tissue. **(A)Negative control**: another view in striatum showing average neurons (black arrow), average glial cells (blue arrow), and average blood vessels (red arrow) (H&E X 400). **(B) Positive control**: another view in striatum showing scattered degenerated neurons (black arrow), average glial cells (blue arrow), and mildly congested blood vessels (red arrow) (H&E X 400). **(C) SP 1**: high power view showing average neurons (black arrow), average glial cells (blue arrow) and average intra-cerebral blood vessels (red arrow), (H&E X 400). **(D) SPN 1**: high power view showing average neurons (black arrow), average glial cells (blue arrow) and average intra-cerebral blood vessels (red arrow), (H&E X 400). **(E) ECH 1**: another view in striatum showing scattered degenerated neurons (black arrow), average glial cells (blue arrow), and average blood vessels (red arrow) (H&E X 400). **(F) ECH + SP**: another view in striatum showing average neurons (black arrow), average glial cells (blue arrow), and average blood vessels (red arrow) (H&E X 400). **(G) ECH + SPN**: high power view showing scattered degenerated neurons (black arrow), average glial cells (blue arrow) and mildly congested intra-cerebral blood vessels (red arrow) (H&E X 400).

**Fig. 12:**
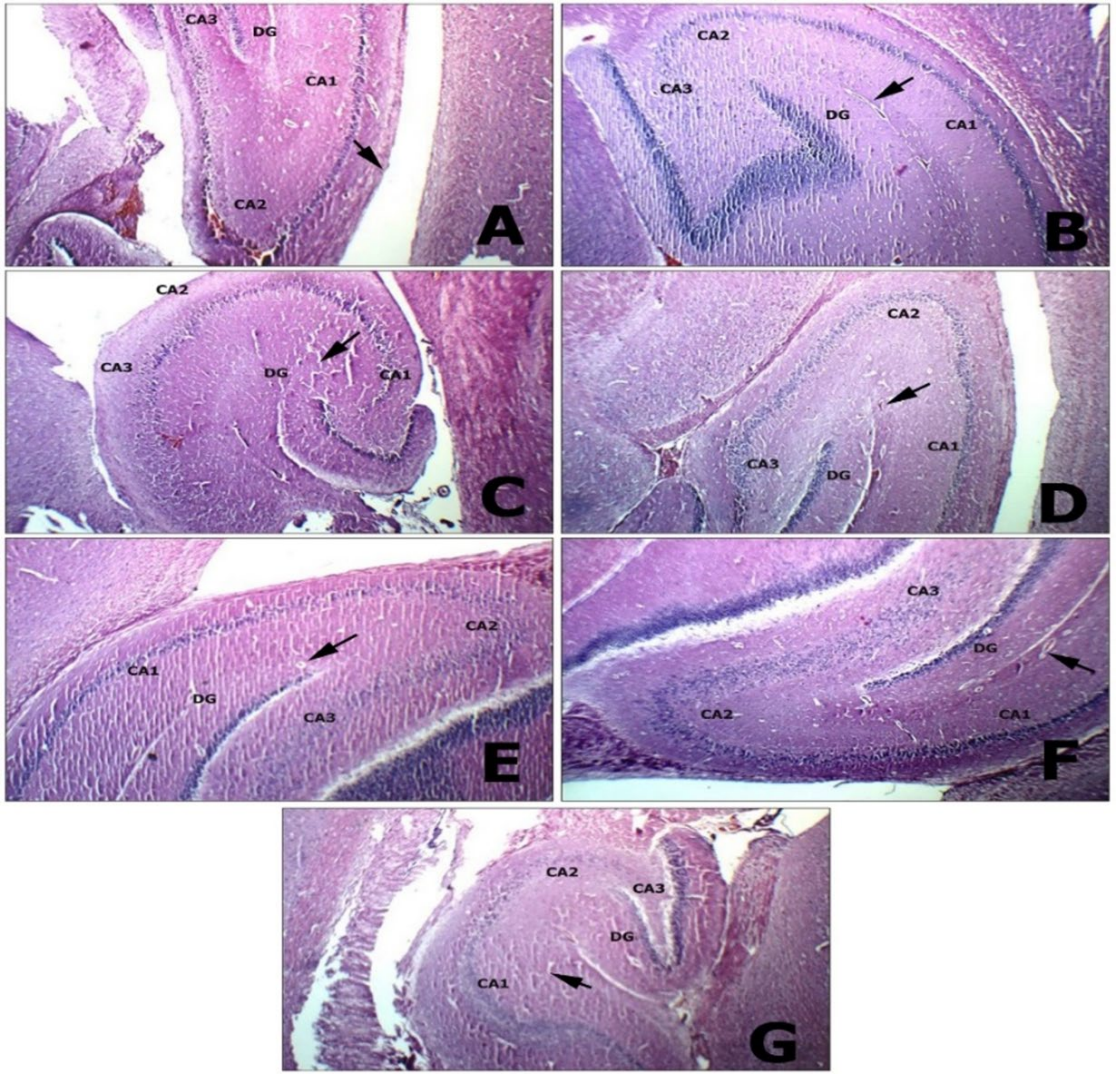
Histopathological investigation of brain tissue at different magnifications (hippocamus). **(A)Negative control**: hippocampus showing average Cornu Amonis (CA1), (CA2), (CA3), average dentate gyrus (DG), and average blood vessels (black arrow) (H&E X 100). **(B) Positive control**: hippocampus showing average Cornu Amonis (CA1), (CA2), (CA3), and average blood vessels (black arrow) (H&E X 100). **(C) SP 1**: hippocampus showing average Cornu Amonis (CA1), (CA2), (CA3), and average blood vessels (black arrow) (H&E X 100). **(D) SPN 1**: hippocampus showing average Cornu Amonis (CA1), (CA2), (CA3), and average blood vessels (black arrow) (H&E X 100). **(E) ECH 1**: hippocampus showing average Cornu Amonis (CA1), (CA2), (CA3), and average blood vessels (black arrow) (H&E X 100). **(F) ECH + SP**: hippocampus showing average Cornu Amonis (CA1), (CA2), (CA3), and average blood vessels (black arrow) (H&E X 100). **(G) ECH + SPN**: hippocampus showing average Cornu Amonis (CA1), (CA2), (CA3), and average blood vessels (black arrow) (H&E X 100).

About the histopathological investigation in different body organs; spleen, heart, and brain in micro and macro investigation at different magnifications as shown in Figs. 9, 10, 11 and 12.

#### Spleen tissue

Spleen showing average capsule, average lymphoid follicles (white bulb) with central arterioles, average blood sinusoids, and average blood vessels. The spleen of CP group in the current study showed atrophied lymphoid follicles with marked hyalinosis, a markedly expanded and congested red bulb with excess ciderophages, and dilated and congested blood vessels. While SP and ECH therapy attenuated the effects of CP, the spleen showed a small number of lymphoid follicles with central arterioles, average lymphocytes in the periarteriolar area, a peri-arteriolar area, a large number of lymphocytes, a mild congested blood vessel, and a small number of lymphocytes scattered throughout the red bulb (Fig. 9).

#### Cardiac tissue

Histological examination of negative control group was showed intact pericardium, viable cardiac muscle fibers with central oval\elongated nuclei, and average myocardial blood vessels. In contrast the CP positive group had intact pericardium of heart, dilated congested blood vessels, markedly apoptotic cardiac muscle fibers, and others with small pyridonal nuclei and large cytoplasmic vacuoles. In SP group, the cardiac tissue has the same cardiac histological picture of the control negative group, while in SPN group scattered apoptotic cardiac muscle fibers, and mildly congested blood vessels were detected. The scattered apoptotic cardiac muscle fibers and others with small cytoplasmic vacuoles, and mildly dilated blood vessels were observed in ECH and Ech/SP group. In the Ech/SPN combination group the average intervening capillaries were noted (Fig. 10).

#### Brain tissue

A histopathological examination of the brain in a CP-intoxicated group revealed scattered degenerated neurons in the striatum, mildly congested blood vessels, and scattered degenerated pyramidal neurons. Results from SP group are nearly identical to those from control groups, as indicated by average meninges and submeningeal blood vessels in the brain, average neurons in the cerebral cortex, average glial cells and intra-cerebral blood vessels in the cerebral cortex, and average neurons, average glial cells, and average blood vessels in the striatum. In the hippocampal area, pyramidal neurons, interneuron area, and blood vessels were all on par. ECH group showed similar results except for mildly congested intra-cerebral blood vessels (Figs. 11 and 12).

## Discussion

The active pharmaceutical compounds could be loaded efficiently on the nanocarriers^48^. Niosome are a multilamellar carrier for both hydrophilic and lipophilic bioactive constituents^49^. Based on a literature review, the optimal SPN was selected for in vitro and in vivo characterization^50^. Tween 60 was chosen as a non-ionic surfactant due to its long alkyl chains, enabling the formation of small niosome with high Entrapment Efficiency (EE) and rigid vesicular membranes^51^. Moreover, the water-soluble SP was better encapsulated by Tween 60 due to its hydrophilicity and high hydrophilic–lipophilic balance (HLB)^52,53^. Their results were similar to those of Waddad et al.^54^. Cholesterol enhances the stability of niosomal vesicles and increases entrapment efficiency. Cholesterol: Tween 60 ratio of 2:1 reduced particle size and increased %EE by lowering surface free energy and enhancing bilayer hydrophobicity^55^. Similar findings were reported by Chaw and Kim^55^. DDP was incorporated to maintain a slightly negative zeta potential, improving niosome stability^52^. Based on the literature, the optimal formulation consisted of Tween 60, cholesterol, and DDP at a molar ratio of 1:2:0.1^54^. Zeta potential is a key parameter for determining nanoparticle surface charge and predicting stability; higher values indicate stronger electrostatic repulsion and improved stability^56^. The SPN formulation showed a uniform particle size distribution with a low polydispersity index (PDI = 0.405), indicating homogeneity without aggregation. Its zeta potential was − 23.2 ± 0.36 mV, where the negative charge promotes electrostatic repulsion between vesicles, enhancing stability^57^.

As compared to control negative group and CP-intoxicated group, SP, SPN, Ech/SP, and Ech/SPN treated groups had significantly higher body weights on the 7th and 15th days. Mohamed et al.^58^ reported that dietary supplementing with EP improves growth performance, intestinal histology, and antioxidant status in mice. Several studies have also demonstrated that EP enhances body weight gain in broilers^59,60^. Furthermore, Nakata et al.^61^ found that dietary Spirulina supplementation increased body weight gain at daily doses ranging from 8.21 to 22, with a linear increase in feed conversion ratio (FCR) from days 8 to 21.

CP-injected rats in the present study showed reductions in RBCs, hemoglobin, and HCT, indicating CP-induced anemia. These findings are consistent with Cengiz^62^, who reported that intraperitoneal administration of CP decreased erythrocytes, hemoglobin, leukocytes, thrombocytes, hematocrit, and bone marrow cells^63^.

In our study, SP, SPN, ECH, ECH/SP, and ECH/SPN treatment improved all hematological parameters in CP-injected rats compared with the untreated CP group, with ECH/SPN showing greater improvement than EP. This effect may be attributed to the bioactive components of EP, including cichoric acid, echinacin, and vitamin E, which stimulate hematopoiesis. Farag et al.^65^ also reported that SP administration enhances iron and hemoglobin metabolisms. Additionally, SP contains minerals, phytonutrients, antioxidants, and polysaccharides, that activate enzymes, boost immune responses at both molecular and humoral levels, and scavenge free radicals^66^. CP is known to induce severe cardiotoxicity^67^. The incidence of CP-induced cardiotoxicity increases with the administered dose^68^. High doses can lead to acute cardiac complications, including the release of toxic metabolites, myocardial injury, and impaired diastolic function^69^. Common manifestations include tachyarrhythmias, myocarditis, hypotension, pericardial disease, and heart failure. Acute heart failure has been reported in 7–33% of cases at doses exceeding 150 mg/kg, as noted by Morandi et al.^70^ and Shanholtz^71^.

CP treatment significantly increased serum CK-MB levels, accompanied by histological changes such as myofibril degeneration, fragmentation, and nuclear pyknosis. Similar elevations in CK-MB following CP administration have been reported by Omole et al.^72^ and El-Sheikh et al.^73^. Cardiac biomarkers, including CK-MB, AST, and IMA, were also elevated in CP-treated groups^74^. Khanra et al.^75^ attributed this increase to enzyme release from necrotic and degenerating cardiac tissue. Therefore, monitoring biomarkers such as CK-MB and AST is essential for assessing cardiac injury severity^76^. Studies by El-Agamy et al.^77^ and Gunes et al.^78^ further confirmed the cardiotoxic effects of CP, including endothelial dysfunction and cardiomyocyte damage. CP also elevates CK-MB levels by inducing lipid peroxidation, which increases endothelial permeability^74^. Administration of *Spirulina* and *Echinacea* with CP significantly reduced serum CK-MB levels compared to CP alone. These findings highlight their potential as adjunctive therapies to mitigate CP-induced cardiotoxicity, as reduced CK-MB levels may reflect decreased cardiac stress.

Prior administration of SP reduced cardiac injury biomarkers and lipid peroxidation in heart tissues, while increasing antioxidant enzymes and glutathione levels in a dose-dependent manner. SP contains potent antioxidants, including phycocyanin, carotene, vitamins, minerals, proteins, lipids, and carbohydrates^79^. Several studies have confirmed the cardioprotective effects of SP and its bioactive components against pharmacological, chemical, and xenobiotic-induced cardiac damage^80^.

EP also exhibits protective effects. The roots of *Echinacea purpurea* possess antioxidants and free radical scavenging properties^81^, largely attributed to polyphenolic components, such as flavonoids, phenolic acids, and phenolic diterpenes^82^. Olama et al.^83^‏ demonstrated that *Echinacea* hosts endophytic fungi capable of producing bioactive compounds, highlighting its dual role as a therapeutic plant and a source of beneficial microorganisms. Elshahawy et al.^84^ further supported the potential of *Echinacea* for producing antioxidant phytochemicals. Additionally, Hu and Kitts^85^ demonstrated that flavonoids and rosmarinic acid effectively scavenge DPPH radicals, while cichoric acid enhances antioxidant activity through improved surface interactions. Treatment of CP-injected rats with *Echinacea purpurea* reduced MDA levels and increased GSH levels and CAT activity, as reported by Mahmoud et al.^86^. EP contains a multitude of bioactive compounds with antioxidant and anti-inflammatory properties^87^. Gargouri et al.^88^ reported that SP reduced lead-induced brain damage in newborn rats, while Abdelmonem et al.^89^ found that *Echinacea* alleviates cardiac inflammation by reducing oxidative stress and promoting cardiac tissue regeneration and survival.

NF-κB is a key regulator of inflammation, controlling the expression of target genes such as COX-2, chemokines, and cytokines^90^. CP-treated groups showed significantly elevated NF-κB expression. CP is known to suppress immune function by inhibiting T and B cell proliferation and differentiation, leading to impaired humoral and cellular responses, along with adverse effects such as bone marrow suppression, hepatotoxicity, mutagenicity, teratogenicity, and carcinogenicity, as reported by Ren et al.^91^. In contrast, NF-κB expression was markedly reduced in groups treated with ECH, SP, their combination (Ech/SP), and nano-formulations (SPN and Ech/SPN), likely due to their protective effects, consistent with Roman-Blas and Jimenez^92^. According to Majai et al.^93^ Ech stimulates phagocytosis, induces cytokine production, and modulates immune cell populations. According to Qi et al.^94^ the reduced cytokine levels in treated groups may also be linked to improved T-cell proliferation. Similarly, Vieira et al.^95^ concluded that *Echinacea purpurea* extracts have been shown to suppress pro-inflammatory mediators, supporting their anti-inflammatory activity. In addition, alkylamides inhibit inflammatory signaling pathways such as extracellular signal-regulated kinase (ERK1/2), p38, NF-κB, and Signal transducer and activator of transcription 3 (STAT3), and down regulate cyclooxygenase-2 (COX-2) expression. Sloley et al.^96^ proposed that the antioxidant characteristics of *Echinacea* extract, including its flavonoids and polyphenolic complexes, have a role in the efficiency of *Echinacea* extract in reducing histopathological alterations.

The spleen of CP in the current study showed severe histopathological lesions including atrophied lymphoid follicles, marked hyalinosis, expanded and congested red bulbs with excess ciderophages, and dilated and congested blood vessels, in agreements with Al-Salih et al.^97^ results. CP, a potent anticancer and immunosuppressive agent, targets lymphocytes and induces their death^98^. In contrast, the administration of SP or *Echinacea* to CP-intoxicated rats in the present work improved all indicators of splenic function and effectively prevented histopathological alterations. Previous data^17^, also showed that *Echinacea* reduced splenic alterations, including red pulp hypocellularity, depletion of periarteriolar lymphoid sheaths, and congestion associated with cisplatin-induced immunotoxicity. As a result, the splenic vein and blood sinusoids were reduced in size and congested, hemosiderin deposition was reduced, and tissue architecture was restored. EP extract may exhibit antioxidant properties due to the presence of various constituents (e.g., polysaccharides and flavonoids) in the spleen.

In our study, the CP positive group had intact pericardium of heart, dilated congested blood vessels, markedly apoptotic cardiac muscle fibers, and others with small pyridonal nuclei and large cytoplasmic vacuoles. Similarly, Motawi et al.^99^ reported hemorrhagic lesions in the myocardium and degeneration of myocardial fibers. These findings are consistent with previous studies, supporting the current results. Cetik et al.^100^ also attributed such tissue damage to membrane injury caused by CP metabolites. These pathological changes were in agreement with alterations in enzyme activity. CP-induced cardiotoxicity is likely mediated by mitochondrial dysfunction, leading to reduced ATP production due to oxidative and nitrosative stress. In contrast, co-administration of ECH and SP with CP reduced tissue damage and histopathological abnormalities such as necrosis, indicating a protective effect on cardiac tissue. This suggests that ECH and SP act as antioxidants and membrane stabilizers, preserving cellular and tissue integrity. CP-induced cardiotoxicity was also found to be dose-dependent, as evidenced by hemorrhage, inflammatory cell infiltration, and separation of myocardial fibers, which supported the biochemical findings. Similarly, pre-treatment with ECH extract before DMBA administration showed marked improvement, characterized by well-organized muscle fibers with clear striations and well-distributed connective tissue^101^.

The histopathological findings in the brain of the CP-intoxicated group were consistent with those reported by Elsayed et al.^102^, showing congested blood vessels, neuronal shrinkage, neurodegeneration, and glial cell proliferation. Marked loss of Purkinje neurons was also observed in the hippocampus and cerebellum. Oxidative stress plays a major role in CP-induced neurotoxicity. Oboh and Ogunruku^103^ reported that CP causes mitochondrial damage in the hippocampus, leading to excessive reactive oxygen species (ROS) production^104^. This oxidative imbalance disrupts antioxidant defense mechanisms, resulting in neuronal injury and cell death. Several studies assessing oxidative stress markers and endogenous antioxidant proteins further confirmed that CP induces severe oxidative damage and cytotoxicity^100^.

In contrast, SP-treated groups showed nearly normal histological architecture, including average meninges and sub-meningeal blood vessels, normal neuronal and glial cell distribution in the cerebral cortex and striatum, and intact hippocampal structures. Similarly, the EP-treated group showed comparable improvements, except for mild congestion of intra-cerebral blood vessels. Mohamed et al.^105^ reported that alcoholic extracts of EP (250 mg/kg) ameliorated aluminum chloride–induced neurodegeneration by inhibiting cholinesterase activity, restoring oxidative balance, downregulating IL-6 and TNF-α, and improving behavioral outcomes, with corresponding reductions in neuronal degeneration and amyloid plaques. Almeida et al.^106^ also observed increased viable neurons and improved motor function following brain injury. The neuroprotective effects of SP and its active component phycocyanin in acute neurological disorders require further investigation. Bermejo-Bescós et al.^25^ demonstrated that SP extract and phycocyanin C reduce hydroxyl and peroxyl radical formation, inhibit lipid peroxidation, and chelate iron, suggesting potential therapeutic roles in neurodegenerative diseases due to iron-related toxicity.

## Conclusion

The administration of *Echinacea purpura* and *Spirulina platensis* (in its free and niosome-loaded formulations) showed promising potential protective effects against the toxicity triggered by cyclophosphamide. These treatments helped to modulate NF-κB activation and restore antioxidant balance, thereby mitigating the oxidative and inflammatory damage induced by cyclophosphamide. Histopathological analysis further confirmed the protective role of these formulations, showing reduced tissue damage in treated groups. These findings highlight the potential of *Echinacea* and *Spirulina* as adjunct therapies for reducing chemotherapy-induced systemic toxicity, with further studies warranted to optimize their use in clinical settings.

## Electronic Supplementary Material

Below is the link to the electronic supplementary material.

Supplementary Material 1

Supplementary Material 2

Supplementary Material 3

Supplementary Material 4

## Data Availability

All data generated or analysed during this study are included in this published article and its supplementary information files.

## Abbreviations

AlCl_3_: Aluminum chloride
CBC: Complete blood picture
CK-MB: Creatinine kinase
COX-2: Cyclooxygenase-2
CP: Cyclophosphamide
DPPH: 2,2-diphenyl-1-(2,4,6-trinitrophenyl)
ECH: Echinacea
EE: Entrapment Efficiency
EP: Echinacea purpura
ERK: Extracellular signal-regulated kinase
GSH: Glutathione
Hb: Hemoglobin
HCT: Hematocrit
HLB: Hydrophilic-Lipophilic Balance
MCH: Mean corpuscular hemoglobin
MCHC: Mean cell hemoglobin concentration
MCV: Mean corpuscular volume
MDA: Malondialdehyde
NF-κB: Nuclear factor kappa B
NO: Nitric oxide
NO: Nitrogen oxide
PCV: Packed cell volume
RBC: Red blood cell
SOD: Superoxide dismutase
SP: Spirulina platensis
SPN: Spirulina platensis niosome
STAT3: Signal transducer and activator of transcription 3
WBC: White blood cell

## References

[1] Hesketh, P. J. et al. Antiemetics: ASCO guideline update. *J. Clin. Oncol.* **38**, 2782–2797. 10.1200/JCO.20.01296 (2020). 32658626 10.1200/JCO.20.01296

[2] Was, H. et al. Mechanisms of chemotherapy-induced neurotoxicity. *Front. Pharmacol.* **13**, 1–32. 10.3389/fphar.2022.750507 (2022). 10.3389/fphar.2022.750507 PMC8996259 35418856

[3] Cetik Yildiz, S. et al. The protection afforded by kefir against cyclophosphamide induced testicular toxicity in rats by oxidant antioxidant and histopathological evaluations. *Sci. Rep.* **14**(1), 18463 (2024a). 39122852 10.1038/s41598-024-67982-y PMC11316007

[4] Ogino, M. H., Tadi, P. & Cyclophosphamide. StatPearls - *NCBI*; (2021). Http://www.ncbi.nlm.nih.gov/books/NBK553087/, Boo.

[5] Baba, J. et al. Depletion of radio-resistant regulatory T cells enhances antitumor immunity during recovery from lymphopenia. *Blood* **120**, 2417–2427. 10.1182/blood-2012-02-411124 (2012). 22806892 10.1182/blood-2012-02-411124

[6] Molinaro, M. et al. Recent advances on pathophysiology, diagnostic and therapeutic insights in cardiac dysfunction induced by antineoplastic drugs. *BioMed Res. Int.*10.1155/2015/138148 (2015). 26583088 10.1155/2015/138148 PMC4637019

[7] Yeh, E. T. H. et al. Cardiovascular complications of cancer therapy: Diagnosis, pathogenesis, and management. *Circulation* **109**, 3122–3131. 10.1161/01.CIR.0000133187.74800.B9 (2004). 15226229 10.1161/01.CIR.0000133187.74800.B9

[8] Dhesi, S. et al. Cyclophosphamide-Induced Cardiomyopathy. *J. Investig Med. High. Impact Case Rep.* **1**, 232470961348034. 10.1177/2324709613480346 (2013). 10.1177/2324709613480346 PMC4528786 26425570

[9] Shi, D. D. et al. Ginsenoside Rg1 Prevents Chemotherapy-Induced Cognitive Cytokines, Impairment: Associations with Microglia-Mediated Neuroinflammation, and Neuroplasticity. *Mol. Neurobiol.* **56**, 5626–5642 (2019a). 30659419 10.1007/s12035-019-1474-9

[10] Shi, D. D. et al. Chemotherapy-induced cognitive impairment is associated with cytokine dysregulation and disruptions in neuro. *Mol. Neurobiol.* **56**(3), 2234–2243 (2019b). 30008071 10.1007/s12035-018-1224-4

[11] Christie, L. A. et al. Impaired cognitive function and hippocampal neurogenesis following cancer chemotherapy. *Clin. Cancer Res.* **18**(7), 1954–1965 (2012). 22338017 10.1158/1078-0432.CCR-11-2000

[12] Kang, S. et al. Chronic treatment with combined chemotherapeutic agents affects hippocampal micromorphometry and function in mice, independently of neuroinflammation. *Exp. Neurobiol.* **27**, 419–436. 10.5607/en.2018.27.5.419 (2018). 30429651 10.5607/en.2018.27.5.419 PMC6221841

[13] Abdel-Aziz, H. O., Ahmed, G. M., Adly, M. A. & Elsayed, H. M. The possible protective role of *Echinacea* and ginger and both of them on the lung of diabetic male rats: Histological and immunohistochemical study. *Egypt. J. Histol.* **45**, 703–719. 10.21608/ejh.2021.71195.1457 (2022).

[14] Kolev, E. et al. *Echinacea purpurea* for the long-term prevention of viral respiratory tract infections during Covid-19 pandemic: A randomized, open, controlled, exploratory clinical study. *Front. Pharmacol.* **13**, 1–9. 10.3389/fphar.2022.856410 (2022). 10.3389/fphar.2022.856410 PMC9087554 35559249

[15] Wahba, H. A. Protective effect of *Echinacea* (*Echinacea angustifolia*), rosemary (*Rosmarinus officinalis*, L.) and dandelion (*Taraxacum officinal*) powder in Alloxan-diabetic rats. *Qual. Educ. Res. J.* **69**, 1–24 (2022).

[16] Stimpel, M., Proksch, A., Wagner, H. & Lohmann-Matthes, M. L. Macrophage activation and induction of macrophage cytotoxicity by purified polysaccharide fractions from the plant *Echinacea purpurea*. *Infect. Immun.* **46**, 845–849. 10.1128/iai.46.3.845-849.1984 (1984). 6389368 10.1128/iai.46.3.845-849.1984 PMC261624

[17] Khalaf, A. A. et al. Protective effect of *Echinacea purpurea* (Immulant) against cisplatin-induced immunotoxicity in rats. *DARU J. Pharm. Sci.* **27**, 233–241. 10.1007/s40199-019-00265-4 (2019). 10.1007/s40199-019-00265-4 PMC6593030 31134491

[18] Abdelmotaleb, M. M., Elshikh, H. H., Abdel-Aziz, M. M., Elaasser, M. M. & Yosri, M. Evaluation of antibacterial efficacy and phytochemical analysis of *Echinacea purpurea* towards MDR strains with clinical origins.. *Al-Azhar Bull. Sci.* **34**(2), 3 (2023).

[19] Hirahashi, T. et al. Activation of the human innate immune system by *Spirulina*: Augmentation of interferon production and NK cytotoxicity by oral administration of hot water extract of *Spirulina platensis*.. *Int. Immunopharmacol.* **2**, 423–434. 10.1016/S1567-5769(01)00166-7 (2002). 11962722 10.1016/s1567-5769(01)00166-7

[20] Wu, Q. et al. The antioxidant, immunomodulatory, and anti-inflammatory activities of *Spirulina*: An overview.. *Arch. Toxicol.* **90**, 1817–1840 (2016). 27259333 10.1007/s00204-016-1744-5

[21] Zedan, A., Shukry, M., El-Moslemany, A. M., Bahnasy, R. M., Eldamaty, H. S. E., Ibraheim,S. S., … Elolimy, A. (2025). Modulatory role of Spirulina*platensis* in oxidative stress, apoptosis, and gene expression in a rat model of dexamethasone-induced hepatotoxicity. *Frontiers in Pharmacology*, *16*, 1610793. 10.3389/fphar.2025.1610793 PMC12399871 40900828

[22] Sinanoglu, O., Yener, A. N., Ekici, S., Midi, A. & Aksungar, F. B. The protective effects of *Spirulina* in cyclophosphamide induced nephrotoxicity and urotoxicity in rats.. *Urology* **80**(6), 1392.e1-1392.e13926 (2012). 22951000 10.1016/j.urology.2012.06.053

[23] Makhlouf, R. & Makhlouf, I. Evaluation of the effect of *Spirulina* against Gamma irradiation induced oxidative stress and tissue injury in rats.I. *Int. J. Appl. Sci. Eng. Res.* **1** (2), 152–164 (2012).

[24] Gaurav, D., Preet, S. & Dua, K. K. Protective effect of *Spirulina platensis* on cadmium induced renal toxicity in Wistar rats.. *Arch. Appl. Sci. Res.* **2**, 390–397 (2010).

[25] Bermejo-Bescós, P., Piñero-Estrada, E. & Villar del Fresno, Á. M. Neuroprotection by *Spirulina platensis* protean extract and phycocyanin against iron-induced toxicity in SH-SY5Y neuroblastoma cells.. *Toxicol. In Vitro* **22**, 1496–1502. 10.1016/J.TIV.2008.05.004 (2008). 18572379 10.1016/j.tiv.2008.05.004

[26] Abd El-Sadek, M. & Ahmed, E. Novel application of *Spirulina platensis* extract as an alternative to the expensive plant growth regulators on *Capparis cartilaginea* (Decne.).. *Al-Azhar J. Pharm. Sci.* **66**(2), 29–41 (2022).

[27] El-Sayed, H., Rawway, M., Abed, N. N. & Salah El Din, R. A. Antibacterial activity of phenolic extract of *Spirulina platensis* and its structural elucidation of bioactive compound. *Egypt. J. Phycol.* **18**(1), 45–57 (2017).

[28] Bertolin, T. E. et al. Antioxidant effect of phycocyanin on oxidative stress induced with monosodium glutamate in rats. *Braz. Arch. Biol. Technol.* **54**, 733–738. 10.1590/s1516-89132011000400012 (2011).

[29] Karim, K. et al. Niosome: A future of targeted drug delivery systems. *J. Adv. Pharm. Technol. Res.* **1**, 374–380. 10.4103/0110-5558.76435 (2010). 22247876 10.4103/0110-5558.76435 PMC3255404

[30] Nowroozi, F. et al. Effect of surfactant type, cholesterol content and various downsizing methods on the particle size of niosome. *Iran. J. Pharm. Res.* **17**, 1–11 (2018). 31011337 PMC6447874

[31] Gamal, A., Saeed, H., Abo El-Ela, F. I. & Salem, H. F. Improving the antitumor activity and bioavailability of sonidegib for the treatment of skin cancer. *Pharmaceutics* **13**, 1–16. 10.3390/pharmaceutics13101560 (2021). 10.3390/pharmaceutics13101560 PMC8537379 34683853

[32] Iqubal, A. et al. Effect of nerolidol on cyclophosphamide-induced bone marrow and hematologic toxicity in Swiss albino mice. *Exp. Hematol.* **82**, 24–32. 10.1016/j.exphem.2020.01.007 (2020). 31987924 10.1016/j.exphem.2020.01.007

[33] Khalil, S. R. et al. *Spirulina platensis* attenuates the associated neurobehavioral and inflammatory response impairments in rats exposed to lead acetate. *Ecotoxicol. Environ. Saf.* **157**, 255–265. 10.1016/J.ECOENV.2018.03.068 (2018). 29625400 10.1016/j.ecoenv.2018.03.068

[34] Zhai, Z. et al. Alcohol extracts of *Echinacea* inhibit production of nitric oxide and tumor necrosis factor-alpha by macrophages in vitro. *Food Agric. Immunol.* **18**, 221–236. 10.1080/09540100701797363 (2007). 18458735 10.1080/09540100701797363 PMC2366993

[35] Sahastrabuddhe, A. P. Counting of RBC and WBC using image processing: A.. *Hemoglobin* **12**(15), 14–17 (2016).

[36] Jain, N. C. Hematologic techniques. In: Jain NC Schalm’sVeterinary hematology. *Lea and Febigen*, *Philadilphia* (1986).

[37] Bull, B. S. Recommendations for reference method for the packed cell volume.. *Lab. Hematol.* **7**, 148 (2001). 12661822

[38] Varley, H. *Practical clinical biochemistry* 5th edn. (William Heinemann Medical Book, 1980).

[39] Würzburg, U. et al. Quantitative determination of creatine kinase isoenzyme catalytic concentrations in serum using immunological methods.. *J. Clin. Chem. Clin. Biochem.* **15**(3), 131–137 (1977). 859004 10.1515/cclm.1977.15.1-12.131

[40] Aebi, H. Catalase in vitro. *Methods Enzym* ;**105**, 121–126. (1984). 10.1016/s0076-6879(84)05016-3 6727660

[41] Paglia, D. E. & Valentine, W. N. Studies on the quantitative and qualitative characterization of erythrocyte glutathione peroxidase.. *J. Lab. Clin. Med.* **70**(1), 158–169 (1967). 6066618

[42] Nishikimi, M., Appaji Rao, N. & Yagi, K. The occurrence of superoxide anion in the reaction of reduced phenazine methosulfate and molecular oxygen. *Biochem. Biophys. Res. Commun.* **46**, 849–854. 10.1016/S0006-291X(72)80218-3 (1972). 4400444 10.1016/s0006-291x(72)80218-3

[43] Beutler, E., Duron, O. & Kelly, B. M. Improved method for the determination of blood glutathione. *J. Lab. Clin. Med.* **61**, 882–888 (1963). 13967893

[44] Green, L. C. et al. Analysis of nitrate, nitrite, and [15N] nitrate in biological fluids.. *Anal. Biochem.* **126**, 131–138. 10.1016/0003-2697(82)90118-X (1982). 7181105 10.1016/0003-2697(82)90118-x

[45] Kir, H. M., Dillioglugil, M. O., Tugay, M. & Eraldemir, C. O. H. Effects of vitamins E, A and D on MDA, GSH, NO levels and SOD activities in 5/6 nephrectomized rats.. *Am. J. Nephrol.* **25**(5), 441–446 (2005). 16118481 10.1159/000087825

[46] Brouckaert, P. et al. Tumor necrosis factor, its receptors and the connection with interleukin 1 and interleukin 6. *Immunobiology*, **187**, 317–329. 10.1016/S0171-2985(11)80347-5 (1993). 8392490 10.1016/S0171-2985(11)80347-5

[47] Hosseini, S. M. et al. Chronic zinc oxide nanoparticles exposure produces hepatic and pancreatic impairment in female rats. *Iran. J. Toxicol.* **14**, 145–154. 10.32598/ijt.14.3.626.1 (2020).

[48] Patra, J. K. et al. Nano based drug delivery systems: Recent developments and future prospects. *J. Nanobiotechnol.* **16**, 1–33. 10.1186/s12951-018-0392-8 (2018). 10.1186/s12951-018-0392-8 PMC6145203 30231877

[49] Moammeri, A. et al. Current advances in niosome applications for drug delivery and cancer treatment. *Mater. Today Bio*. **23**, 100837. 10.1016/j.mtbio.2023.100837 (2023). 37953758 10.1016/j.mtbio.2023.100837 PMC10632535

[50] Chauhan, M. K. Bioavailability enhancement of polymyxin B with novel drug delivery: Development and optimization using quality-by-design approach. *J. Pharm. Sci.* **108**(4), 1521–1528 (2019). 30472265 10.1016/j.xphs.2018.11.032

[51] Abdelbary, G. & El-Gendy, N. Niosome-encapsulated gentamicin for ophthalmic controlled delivery. *AAPS PharmSciTech* **9**, 740–747. 10.1208/s12249-008-9105-1 (2008). 18563578 10.1208/s12249-008-9105-1 PMC2977028

[52] Bnyan, R. et al. Surfactant effects on lipid-based vesicles properties. *J. Pharm. Sci.* **107**(5), 1237–1246 (2018). 29336980 10.1016/j.xphs.2018.01.005

[53] Nowroozi, F. et al. Effect of surfactant type, cholesterol content and various downsizing methods on the particle size of niosome. *Iran. J. Pharm. Res.* **17**, 1–11 (2018). 31011337 PMC6447874

[54] Waddad, A. Y. et al. Formulation, characterization and pharmacokinetics of Morin hydrate niosome prepared from various non-ionic surfactants. *Int. J. Pharm.* **456**, 446–458. 10.1016/J.IJPHARM.2013.08.040 (2013). 23998955 10.1016/j.ijpharm.2013.08.040

[55] Chaw, C. S. & Kim, K. Y. Effect of formulation compositions on niosomal preparations. *Pharm. Dev. Technol.* **18** (3), 667–672 (2013). 22468904 10.3109/10837450.2012.672988

[56] Moustafa, D., Mahmoud, R., El-Salam, H. M. A. & Shehata, N. Utilization of residual zinc–iron-layered double hydroxide after methyl orange management as a new sorbent for wastewater treatment. *Appl. Nanosci.* **11**, 709–723. 10.1007/s13204-020-01632-3 (2021).

[57] Al Shuwaili, A. H., Rasool, B. K. A. & Abdulrasool, A. A. Optimization of elastic transfersomes formulations for transdermal delivery of pentoxifylline. *Eur. J. Pharm. Biopharm.* **102**, 101–114. 10.1016/J.EJPB.2016.02.013 (2016). 26925505 10.1016/j.ejpb.2016.02.013

[58] Mohamed, N. A., Hashem, M. A. M., Alzahrani, A. M., Abdel-Moneim, A. M. & Abdou, H. M. Hepatoprotective effect of *Spirulina platensis* against carbon tetrachloride-induced liver injury in male rats. *J. Pharm. Pharmacol.* **73**(11), 1562–1570 (2021). 34387320 10.1093/jpp/rgab107

[59] Hashem, M. A., Neamat-Allah, A. N. F., Hammza, H. E. E. & Abou-Elnaga, H. M. Impact of dietary supplementation with *Echinacea* purpurea on growth performance, immunological, biochemical, and pathological findings in broiler chickens infected by pathogenic E. coli. *Trop. Anim. Health Prod.* **52**(4), 1599–1607 (2020). 31828572 10.1007/s11250-019-02162-z

[60] Rady, W. F., Sayed, A. B. N. & Abdel-Raheem, H. A. Effect of dietary supplementation of *Echinacea* and nucleotides on productive performance, intestinal histomorphology and gene expression of broiler chickens. *Assiut Vet. Med. J.* **69**, 141–155. 10.21608/AVMJ.2023.185576.1115 (2023).

[61] Nakata, H. et al. Evaluation of the ameliorative effect of *Spirulina* (Arthrospira platensis) supplementation on parameters relating to lead poisoning and obesity in C57BL/6J mice. *J. Funct. Foods* **77**, 104344. 10.1016/j.jff.2020.104344 (2021).

[62] Cengiz, M. Hematoprotective effect of boron on cyclophosphamide toxicity in rats. *Cell. Mol. Biol.* **64** (5), 62–65 (2018). 29729695

[63] Cengiz, M., Ayhancı, A. & Kutlu, H. M. Investigation into the protective effects of escin on blood cells and cyclophosphamide-induced bone marrow toxicity in rats. *Bilecik Şeyh Edebali Üniv. Fen Bil. Derg.* **7**(2), 730–738 (2020).

[64] Goel, V. et al. Alkylamides of *Echinacea purpurea* stimulate alveolar macrophage function in normal rats. *Int. Immunopharmacol.* **2**, 381–387. 10.1016/S1567-5769(01)00163-1 (2002). 11811940 10.1016/s1567-5769(01)00163-1

[65] Farag, M. R., Alagawany, M., El-Hack, M. E. A. & Dhama, K. Nutritional and healthical aspects of *Spirulina* (Arthrospira) for poultry, animals and human. *Int. J. Pharmacol.* **12**, 36–51. 10.3923/ijp.2016.36.51 (2016).

[66] Viswanadha, V. P., Sivan, S. & Rajendra Shenoi, R. Protective effect of *Spirulina* against 4-nitroquinoline-1-oxide induced toxicity. *Mol. Biol. Rep.* **38**, 309–317. 10.1007/s11033-010-0109-z (2011). 20352348 10.1007/s11033-010-0109-z

[67] Cetik Yildiz, S. et al. In vitro antitumor and antioxidant capacity as well as ameliorative effects of fermented kefir on cyclophosphamide-induced toxicity on cardiac and hepatic tissues in rats. *Biomedicines* **12**(6), 1199 (2024b). 38927407 10.3390/biomedicines12061199 PMC11200811

[68] Iqubal, A. et al. Molecular mechanism involved in cyclophosphamide-induced cardiotoxicity: Old drug with a new vision. *Life Sci.* **218**, 112–131. 10.1016/J.LFS.2018.12.018 (2019). 30552952 10.1016/j.lfs.2018.12.018

[69] Kurauchi, K. et al. Role of metabolites of cyclophosphamide in cardiotoxicity. *BMC Res. Notes.* **10**, 1–10. 10.1186/s13104-017-2726-2 (2017). 28807058 10.1186/s13104-017-2726-2 PMC5557551

[70] Morandi, P. et al. Serum cardiac troponin I levels and ECG/Echo monitoring in breast cancer patients undergoing high-dose (7 g/m2) cyclophosphamide. *Bone Marrow Transplant.* **28**, 277–282 (2001). 11535996 10.1038/sj.bmt.1703132

[71] Shanholtz, C. Acute life-threatening toxicity of cancer treatment. *Crit. Care Clin.* **17**(3), 483–502 (2001). 11529252 10.1016/s0749-0704(05)70196-2

[72] Omole, J. G. et al. Protective Effect of Kolaviron on Cyclophosphamide-Induced Cardiac Toxicity in Rats. *J. Evidence-Based Integr. Med.* **23**, 1–11. 10.1177/2156587218757649 (2018). 10.1177/2156587218757649 PMC5871040 29468886

[73] El-Sheikh, A. A., Abdelzaher, W. Y., Gad, A. A. & Abdel-Gaber, S. A. Purine versus non-purine xanthine oxidase inhibitors against cyclophosphamide-induced cardiac and bone marrow toxicity in rats. *Hum. Exp. Toxicol.* **39**, 249–261. 10.1177/0960327119883412 (2020). 31640406 10.1177/0960327119883412

[74] Cengiz, M., Kutlu, H. M., Peker Cengiz, B. & Ayhanci, A. Escin attenuates oxidative damage, apoptosis and lipid peroxidation in a model of cyclophosphamide-induced liver damage. *Drug Chem. Toxicol.* **45**(3), 1180–1187 (2022). 32838567 10.1080/01480545.2020.1810262

[75] Khanra, R., Dewanjee, S. & Dua, K. *Abroma augusta* L. (Malvaceae) leaf extract attenuates diabetes induced nephropathy and cardiomyopathy via inhibition of oxidative stress and inflammatory response. *J. Transl. Med.* **13**, 1–14. 10.1186/s12967-014-0364-1 (2015). 25591455 10.1186/s12967-014-0364-1 PMC4301895

[76] Comba, B., Çinar, A., Comba, A. & Gencer, Y. G. Sıçanlarda ACTH uygulamasının böbrek fonksiyon testleri, elektrolitler ve hematolojik parametreler üzerine etkileri. *Ankara Univ. Vet. Fak. Derg.* **63**, 229–233. 10.1501/Vetfak_0000002734 (2016).

[77] El-Agamy, D. S., Elkablawy, M. A. & Abo-Haded, H. M. Modulation of cyclophosphamide-induced cardiotoxicity by methyl palmitate. *Cancer Chemother. Pharmacol.* **79** (2), 399–409 (2017). 28130575 10.1007/s00280-016-3233-1

[78] Gunes, S., Sahinturk, V., Karasati, P., Sahin, I. K. & Ayhanci, A. Cardioprotective effect of Selenium against Cyclophosphamide-induced cardiotoxicity in rats. *Biol. Trace Elem. Res.* **177**(1), 107–114 (2017). 27709497 10.1007/s12011-016-0858-1

[79] Upasani, C. & Balaraman, R. Protective effect of *Spirulina* on lead induced deleterious changes in the lipid peroxidation and endogenous antioxidants in rats. *Phyther Res. Int. J. Devoted Pharmacol. Toxicol. Eval Nat. Prod. Deriv* **17**(4), 330–334 (2003). 10.1002/ptr.1135 12722134

[80] Khan, M. et al. C-phycocyanin ameliorates doxorubicin-induced oxidative stress and apoptosis in adult rat cardiomyocytes. *J. Cardiovasc. Pharmacol.* **47**, 9–20. 10.1097/01.fjc.0000191520.48404.27 (2006). 16424780 10.1097/01.fjc.0000191520.48404.27

[81] Ali, E. H. Protective effects of *Echinacea* on cyproterone acetate induced liver damage in male rats. *Pakistan J. Biol. Sci.* **11**(21), 2464–2471 (2008). 10.3923/pjbs.2008.2464.2471 19205265

[82] Stanisavljević, I. et al. Antioxidant and antimicrobial activities of *Echinacea (Echinacea purpurea L.)* extracts obtained by classical and ultrasound extraction. *Chin. J. Chem. Eng.* **17**, 478–483. 10.1016/S1004-9541(08)60234-7 (2009).

[83] Olama, S. M., Sheata, R., Shiekh, E., Younis, A. M. & H., & Phytochemistry and biodiversity of endophytic fungal metabolites isolated from medicinal plants. *Al-Azhar J. Agricultural Res.* **48** (2), 42–58 (2023).

[84] Elshahawy, O. A., Zeawail, M. E. F., Hamza, M. A., Elateeq, A. A. & Omar, M. A. Improving the production of total phenolics and flavonoids and the antioxidant capacity of *Echinacea purpurea* callus through biotic elicitation. *Egypt. J. Chem.* **65** (12), 137–149 (2022).

[85] Hu, C. & Kitts, D. D. Studies on the antioxidant activity of *Echinacea* root extract. *J. Agric. food Chem.* **48** (5), 1466–1472 (2000). 10820044 10.1021/jf990677+

[86] Mahmoud, A. H., Abbas, M. M. & Abdelmonem, H. A. The antioxidant effects of Cerium Oxide nanoparticles and *Echinacea purpurea* against lead-induced immunosuppression in male albino rats. *Egypt. J. Hosp. Med.* **89**, 6106–6114. 10.21608/ejhm.2022.268099 (2022).

[87] Aarland, R. C. et al. Studies on phytochemical, antioxidant, anti-inflammatory, hypoglycaemic and antiproliferative activities of *Echinacea purpurea* and *Echinacea angustifolia* extracts. *Pharm. Biol.* **55**, 649–656. 10.1080/13880209.2016.1265989 (2017). 27951745 10.1080/13880209.2016.1265989 PMC6130640

[88] Gargouri, M. et al. *Spirulina* or dandelion-enriched diet of mothers alleviates lead-induced damages in brain and cerebellum of newborn rats. *Food Chem. Toxicol.* **50**, 2303–2310. 10.1016/J.FCT.2012.04.003 (2012). 22504531 10.1016/j.fct.2012.04.003

[89] Abdelmonem, M. et al. Avemar and *Echinacea* extracts enhance mobilization and homing of CD34 + stem cells in rats with acute myocardial infarction. *Stem Cell Res. Ther.* **6**, 1–17. 10.1186/s13287-015-0171-5 (2015). 26369808 10.1186/s13287-015-0171-5 PMC4570476

[90] Abd El Hady Mousa, M., Mansour, H., Eid, F. & Mashaal, A. Anti-inflammatory activity of ginger modulates macrophage activation against the inflammatory pathway of monosodium glutamate. *J. Food Biochem.* **45**(8), e13819 (2021). 10.1111/jfbc.13819 34159624

[91] Ren, Z. et al. Immuno-enhancement effects of ethanol extract from *Cyrtomium macrophyllum* (Makino) Tagawa on cyclophosphamide-induced immunosuppression in BALB/c mice. *J. Ethnopharmacol.* **155**, 769–775. 10.1016/J.JEP.2014.06.021 (2014). 24960181 10.1016/j.jep.2014.06.021

[92] Roman-Blas, J. A. & Jimenez, S. A. NF-κB as a potential therapeutic target in osteoarthritis and rheumatoid arthritis. *Osteoarthr. Cartil.* **14**, 839–848. 10.1016/j.joca.2006.04.008 (2006). 10.1016/j.joca.2006.04.008 16730463

[93] Majai, G. et al. PPARγ-dependent regulation of human macrophages in phagocytosis of apoptotic cells. *Eur. J. Immunol.* **37**, 1343–1354. 10.1002/eji.200636398 (2007). 17407194 10.1002/eji.200636398

[94] Qi, Q. et al. Protective effect of bergenin against cyclophosphamide-induced immunosuppression by immunomodulatory effect and antioxidation in balb/c mice. *Molecules*10.3390/molecules23102668 (2018). 30336565 10.3390/molecules23102668 PMC6222609

[95] Vieira, S. F. et al. *Echinacea purpurea* fractions represent promising plant-based anti-inflammatory formulations. *Antioxidants*, **12**10.3390/antiox12020425 (2023). 10.3390/antiox12020425 PMC9952182 36829986

[96] Sloley, B. D. et al. Comparison of chemical components and antioxidants capacity of different *Echinacea*. *J. Pharm. Pharmacol.* **53**(6), 849–857 (2001). 11428661 10.1211/0022357011776009

[97] Al-Salih, H. A., Al-Sharafi, N. M., Al-Qabi, S. S. & Al-Darwesh, A. A. The pathological features of cyclophosphamide induced multi-organs toxicity in male wister rats. *Syst. Rev. Pharm.* **11**, 45–49. 10.31838/srp.2020.6.10 (2020).

[98] Araghi, R. R. et al. Iterative optimization yields Mcl-1–targeting stapled peptides with selective cytotoxicity to Mcl-1–dependent cancer cells. *Proc. Natl. Acad. Sci. U S A*. **115**, E886–E895. 10.1073/pnas.1712952115 (2018). 29339518 10.1073/pnas.1712952115 PMC5798337

[99] Motawi, T. M. K., Sadik, N. A. H. & Refaat, A. Cytoprotective effects of DL-alpha-lipoic acid or squalene on cyclophosphamide-induced oxidative injury: An experimental study on rat myocardium, testicles and urinary bladder. *Food Chem. Toxicol.* **48**, 2326–2336. 10.1016/J.FCT.2010.05.067 (2010). 20573578 10.1016/j.fct.2010.05.067

[100] Cetik, S., Ayhanci, A. & Sahinturk, V. Protective effect of carvacrol against oxidative stress and heart injury in cyclophosphamide-induced cardiotoxicity in rat. *Brazilian Arch. Biol. Technol.* **58**, 569–576. 10.1590/S1516-8913201500022 (2015).

[101] Salah, E., El esh, H., Abdel-Reheim, E. S. & Abdul-Hamid, M. Ameliorative effects of Artemisia and *Echinacea* extracts against hepato and cardiotoxicity induced by DMBA on albino rats: experimental and molecular docking analyses. *Beni-Suef Univ. J. Basic. Appl. Sci.* 11. 10.1186/s43088-022-00286-0 (2022).

[102] Elsayed, F. F. et al. Ameliorative effect of flavocoxid on cyclophosphamide-induced cardio and neurotoxicity via targeting the GM-CSF/NF-κB signaling pathway. *Environ. Sci. Pollut Res.* **29**, 69635–69651. 10.1007/s11356-022-20441-5 (2022). 10.1007/s11356-022-20441-5 PMC9512761 35576032

[103] Oboh, G. & Ogunruku, O. O. Cyclophosphamide-induced oxidative stress in brain: Protective effect of hot short pepper (Capsicum frutescens L. var. abbreviatum). *Exp. Toxicol. Pathol.* **62**, 227–233. 10.1016/J.ETP.2009.03.011 (2010). 19447589 10.1016/j.etp.2009.03.011

[104] Oyagbemi, A. A. et al. Gallic Acid Ameliorates Cyclophosphamide-Induced Neurotoxicity in Wistar Rats Through Free Radical Scavenging Activity and Improvement in Antioxidant Defense System. *J. Diet. Suppl.* **13** (4), 402–419 (2016). 26716793 10.3109/19390211.2015.1103827

[105] Mohamed, S. M. et al. Evaluation of anti-Alzheimer activity of *Echinacea purpurea* extracts in aluminum chloride-induced neurotoxicity in rat model. *J. Chem. Neuroanat.* **128**, 102234. 10.1016/J.JCHEMNEU.2023.102234 (2023). 36640914 10.1016/j.jchemneu.2023.102234

[106] Almeida, T. et al. Exploring the neuroprotective effects of *Spirulina platensis*: Insights into hemorrhagic volume and histological outcomes.. *Cureus*, **15**, 10.7759/cureus.42078 (2023). 10.7759/cureus.42078 PMC10434819 37602106

