# Supplementary material for: Ameliorative effects of Spirulina platensis niosome and Echinacea purpura on cyclophosphamide-induced splenic, cardiac and neurotoxicity via modulating NF-κB pathway and oxidative stress
Source: Sci Rep. 2026 May 11;16:14726. doi: 10.1038/s41598-026-51198-3 (PMC13161227; doi:10.1038/s41598-026-51198-3)
Supplement: Supplementary file 3 — Supplementary Material 3 [file 41598_2026_51198_MOESM3_ESM.docx]

**Characterization of** **Spirulina Platensis Niosome**
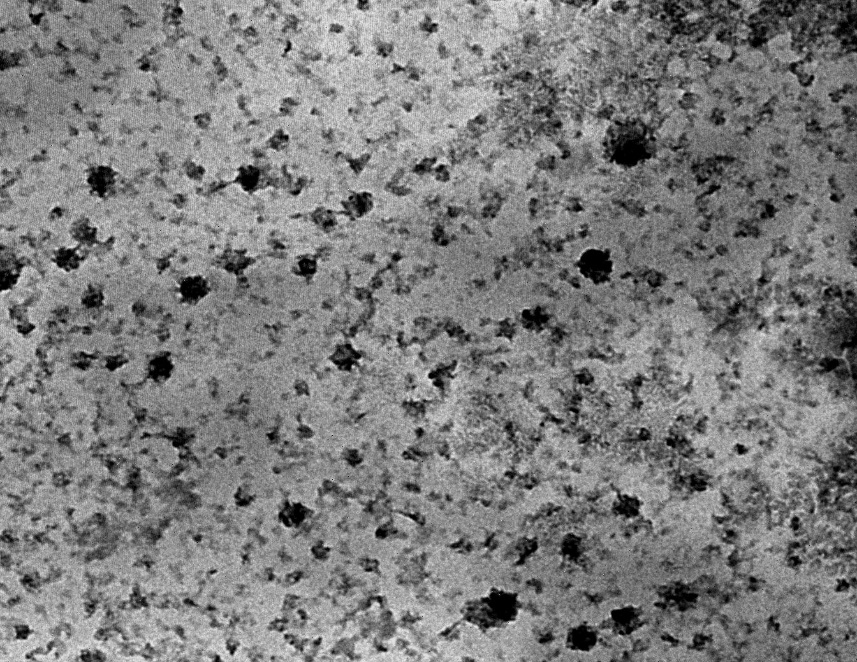


Figure 1 A


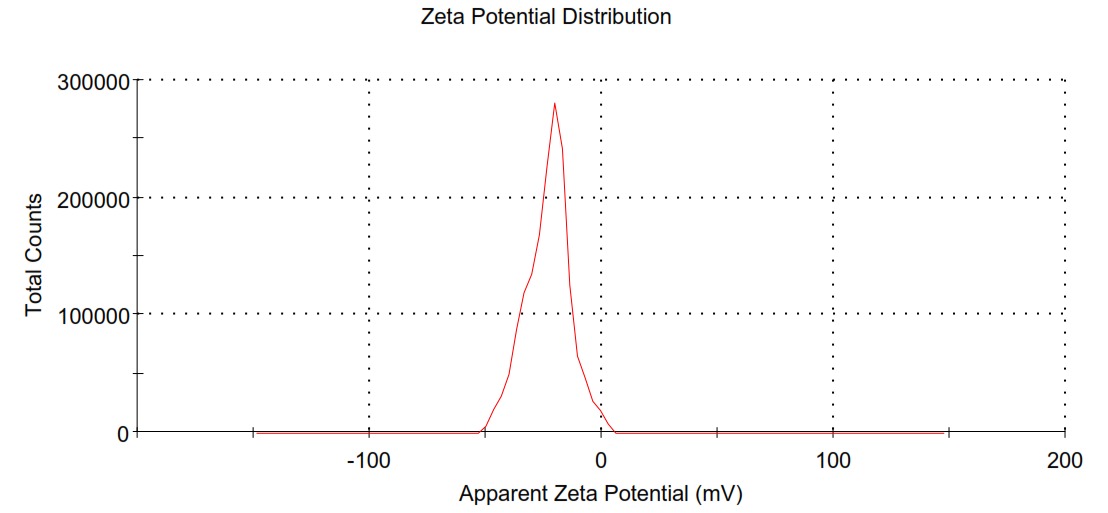


Figure 1 B


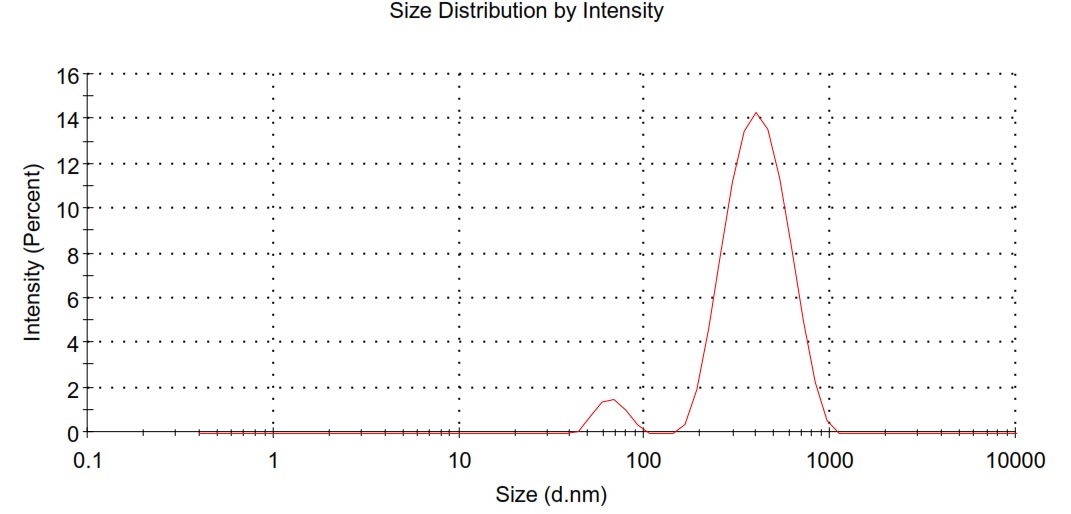


Figure 1 C

**Fig. 1:** Transmission electron microscopy showing obvious SPN formulations without aggregation and successful loading of SP into niosomal Nano carriers (A); Size distribution by intensity of SPN formulations (B); Zeta potential spectrum of the SPN formula (C).
