# Supplementary material for: Ameliorative effects of Spirulina platensis niosome and Echinacea purpura on cyclophosphamide-induced splenic, cardiac and neurotoxicity via modulating NF-κB pathway and oxidative stress
Source: Sci Rep. 2026 May 11;16:14726. doi: 10.1038/s41598-026-51198-3 (PMC13161227; doi:10.1038/s41598-026-51198-3)
Supplement: Supplementary file 4 — Supplementary Material 4 [file 41598_2026_51198_MOESM4_ESM.docx]

**Histopathological Sections**


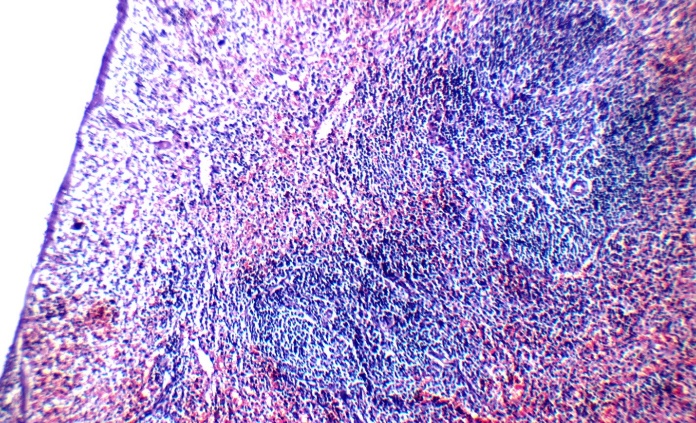

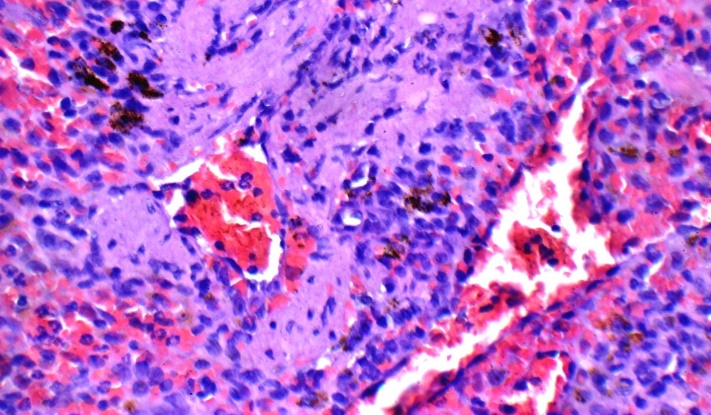


**A**

**B**


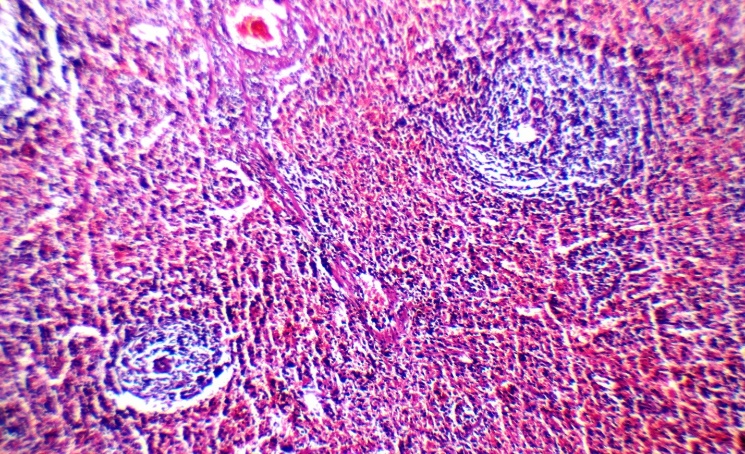

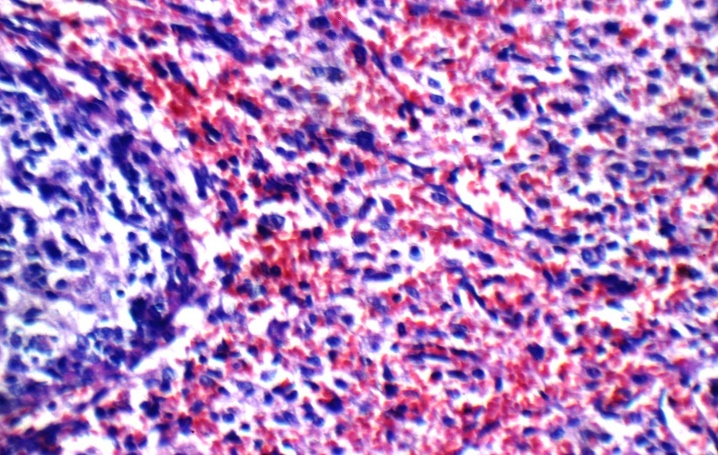


**D**

**C**


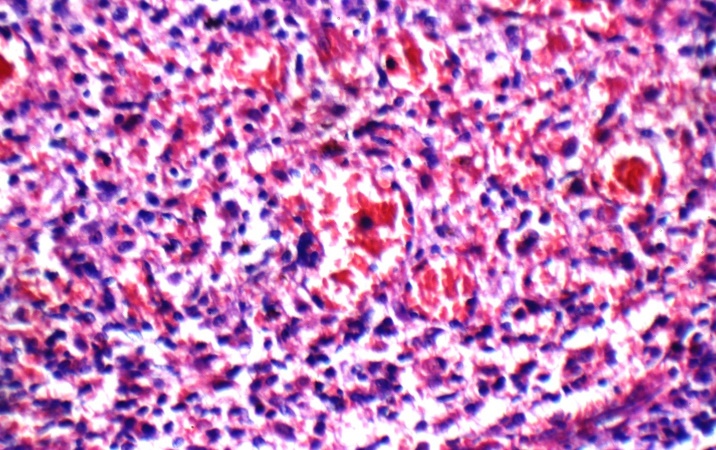

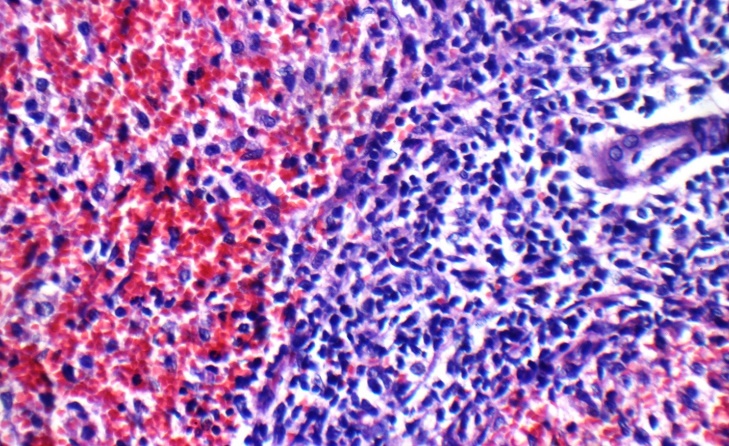


**FF**

**E**


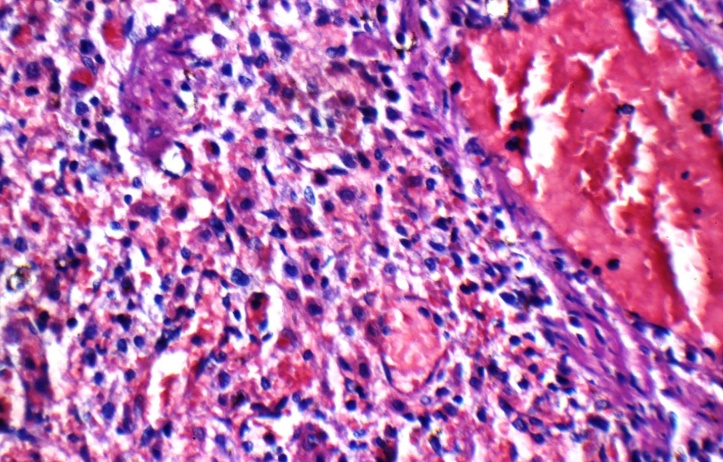

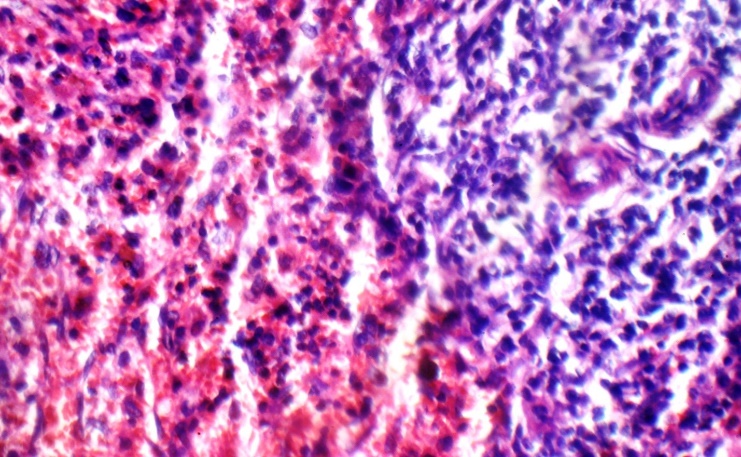


**HF**

**GF**

**Fig. 8: Histopathological investigation of spleen tissue** **(A) Negative control:** spleen showing average capsule (black arrow), average lymphoid follicles (white bulb) with central arterioles (yellow arrow), average blood sinusoids (red bulb) (blue arrow), and average blood vessels (red arrow) (H&E x 200). **(B) Positive control:** another view showing atrophied lymphoid follicles with marked hyalinosis (black arrow), and markedly dilated congested blood vessels (blue arrow) with excess ciderophages (red arrow) (H&E x 400) . **(C) SP 1:** spleen showing small-sized lymphoid follicles with central arterioles (black arrow), expanded (red bulb) (blue arrow), and mildly congested blood vessels (red arrow) (H&E x 200). **(D) SPN 1:** another view showing average blood sinusoids (red bulb) (black arrow), with scattered ciderophages (red arrow) (H&E x 400). **(E) ECH 1:** another view showing average blood sinusoids (red bulb) (black arrow), with scattered ciderophages (red arrow) (H&E x 400). **(F) ECH +SP:** high power view showing small-sized lymphoid follicles with central arterioles (black arrow), and average lymphocytes in peri-arteriolar area (red arrow). **(G) ECH +SPN:** another view showing markedly expanded red bulb with scattered ciderophages (black arrow), and markedly congested blood vessels (red arrow) (H&E x 400). **(H)** **ECH +SPN:** high power view showing atrophied lymphoid follicles with central arterioles (black arrow), and average lymphocytes in peri-arteriolar area (red arrow).


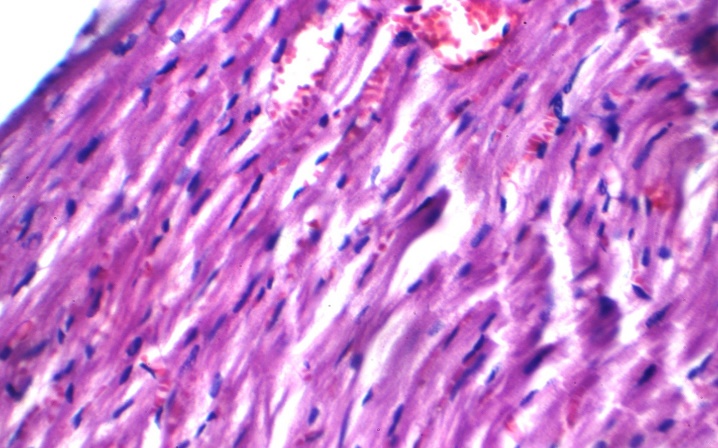

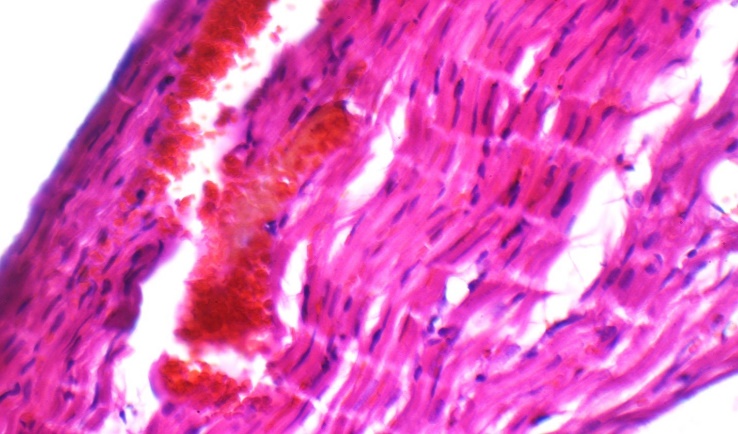


**B**

**A**


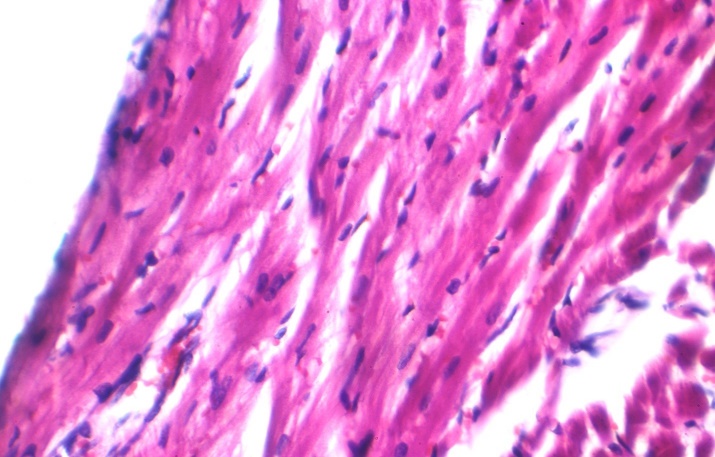

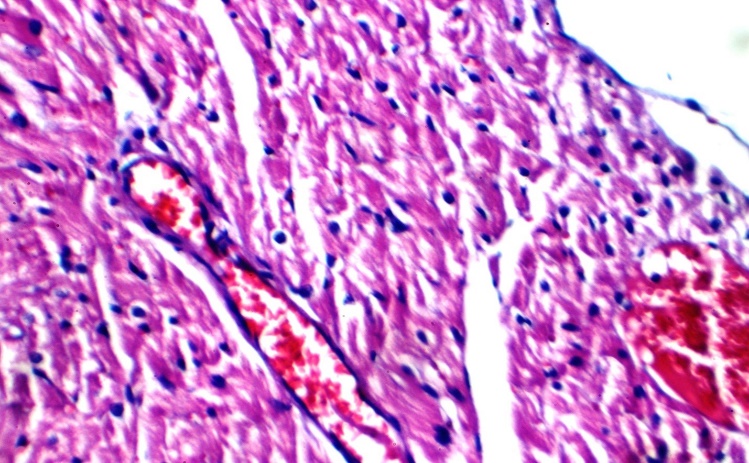


**D**

**C**


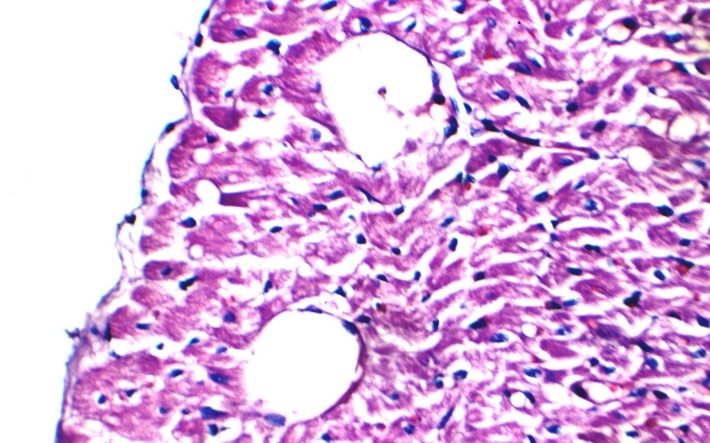

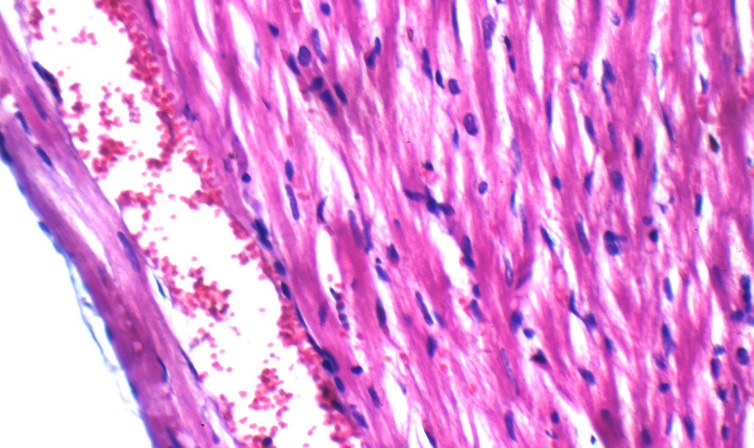


**F**

**E**


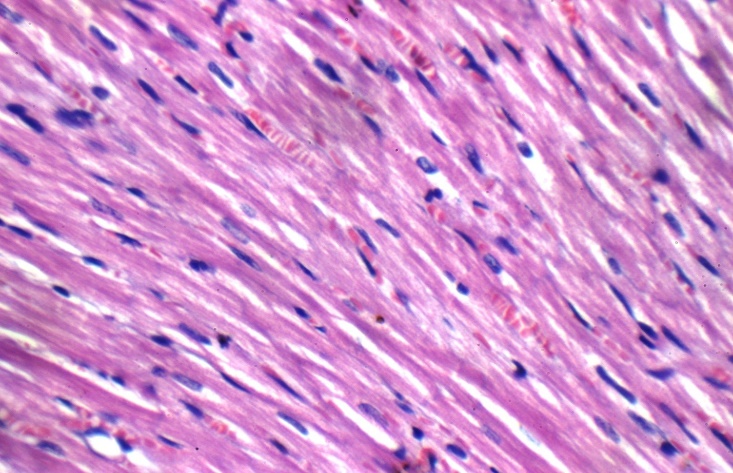


**G**

**Fig. 9: Histopathological investigation of cardiac tissue**

**(A) Negative control:** high power view showing intact pericardium (black arrow), viable cardiac muscle fibers with central oval\elongated nuclei (blue arrow), and average myocardial blood vessels (red arrow) (H&E X 400). **(B) Positive control:** cardiac wall showing intact pericardium (black arrow), markedly apoptotic cardiac muscle fibers (blue arrow), and markedly congested sub-pericardial blood vessels (red arrow) (H&E X 400). **(C) SP 1:** high power view showing intact pericardium (black arrow), viable cardiac muscle fibers with central oval\elongated nuclei (blue arrow), and average blood vessels (red arrow) (H&E X 400). **(D) SPN 1:** high power view showing intact pericardium (black arrow), scattered apoptotic cardiac muscle fibers (blue arrow), and mildly congested blood vessels (red arrow) (H&E X 400). **(E) ECH 1:** high power view showing intact pericardium (black arrow), scattered apoptotic cardiac muscle fibers (blue arrow) and others with small cytoplasmic vacuoles (green arrow), and mildly dilated blood vessels (red arrow) (H&E X 400). **(F) ECH + SP:** high power view showing intact pericardium (black arrow), average cardiac muscle fibers with average nuclei (blue arrow), and mildly dilated blood vessels (red arrow) (H&E X 400). **(G) ECH + SPN:** another view showing average cardiac muscle fibers with average nuclei (black arrow), and average intervening capillaries (red arrow) (H&E X 400).


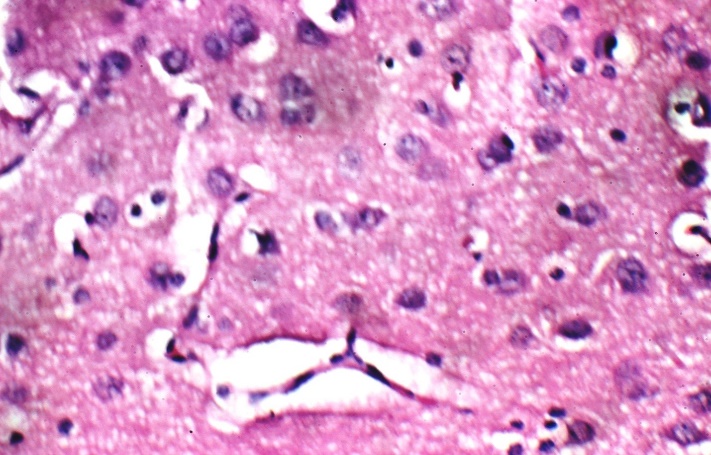

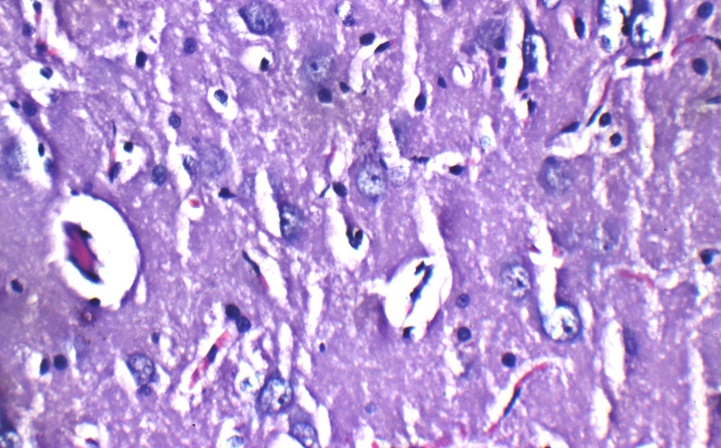


B

A


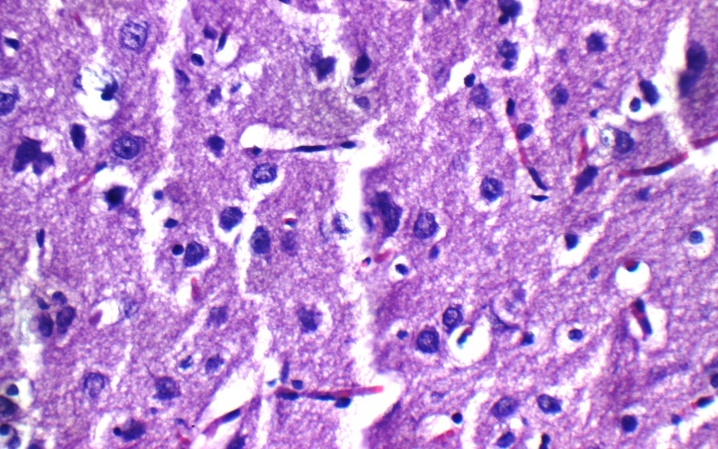

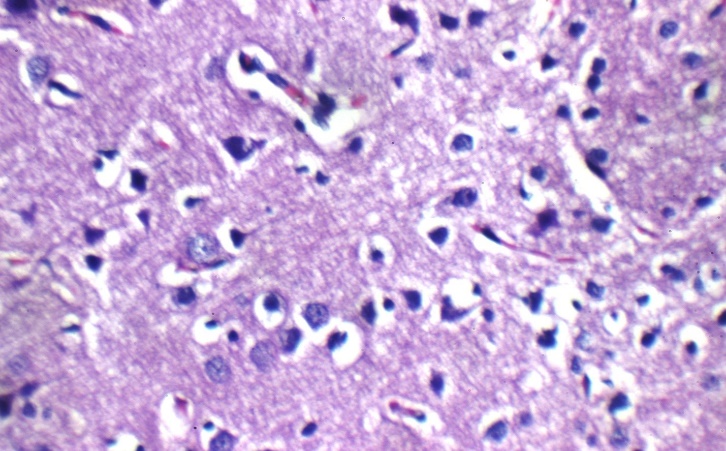


D

C


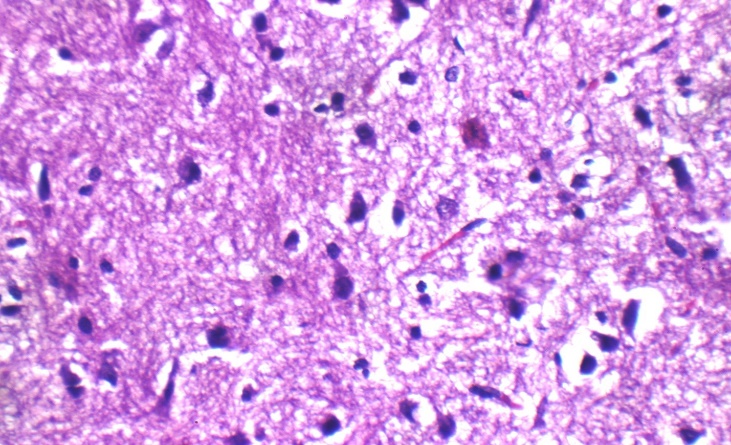

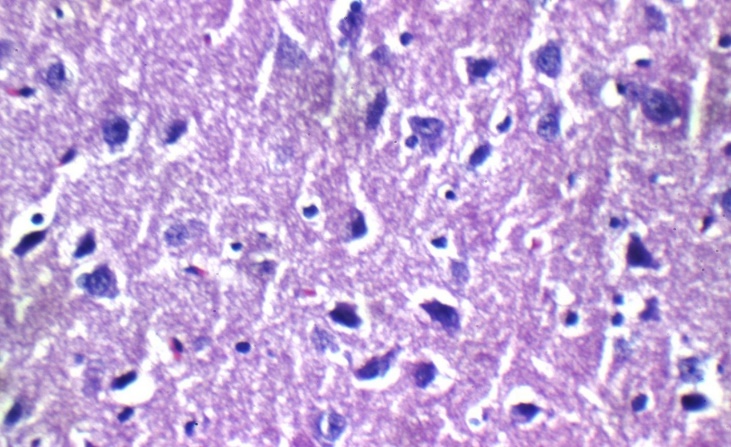


**F**

**E**


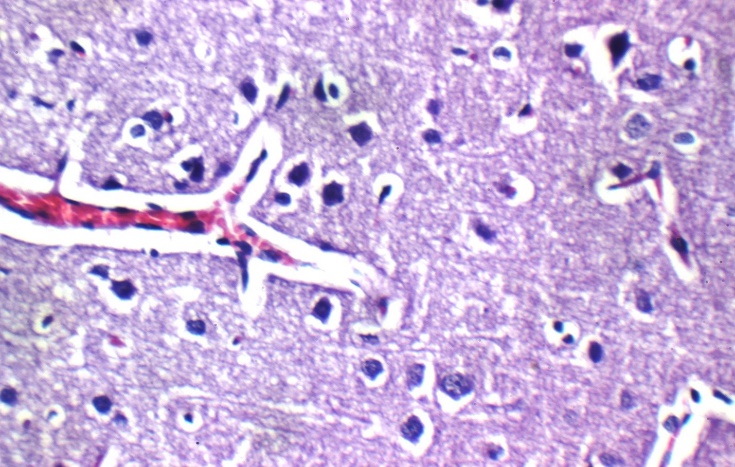


**G**

**Fig. 10: Histopathological investigation of Brain tissue. (A)Negative control:** another view in striatum showing average neurons (black arrow), average glial cells (blue arrow), and average blood vessels (red arrow) (H&E X 400). **(B) Positive control:** another view in striatum showing scattered degenerated neurons (black arrow), average glial cells (blue arrow), and mildly congested blood vessels (red arrow) (H&E X 400). **(C) SP 1:** high power view showing average neurons (black arrow), average glial cells (blue arrow) and average intra-cerebral blood vessels (red arrow), (H&E X 400). **(D) SPN 1:** high power view showing average neurons (black arrow), average glial cells (blue arrow) and average intra-cerebral blood vessels (red arrow), (H&E X 400). **(E) ECH 1:** another view in striatum showing scattered degenerated neurons (black arrow), average glial cells (blue arrow), and average blood vessels (red arrow) (H&E X 400). **(F) ECH + SP:** another view in striatum showing average neurons (black arrow), average glial cells (blue arrow), and average blood vessels (red arrow) (H&E X 400). **(G)** **ECH + SPN:** high power view showing scattered degenerated neurons (black arrow), average glial cells (blue arrow) and mildly congested intra-cerebral blood vessels (red arrow) (H&E X 400).

**A**


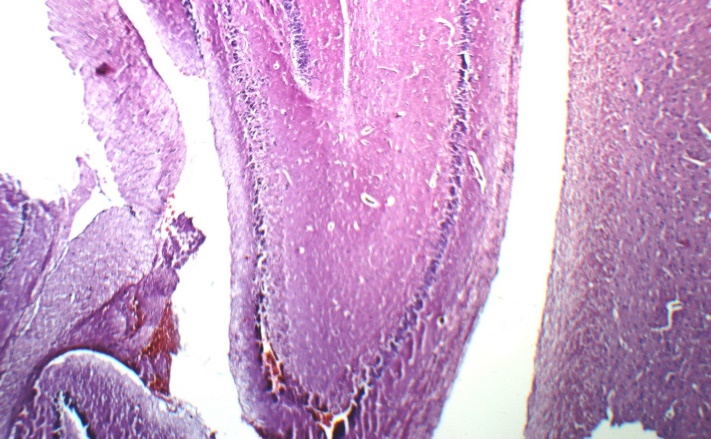

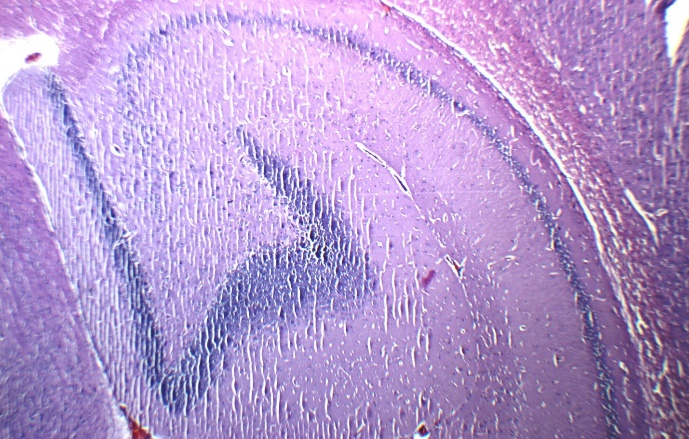


**B**


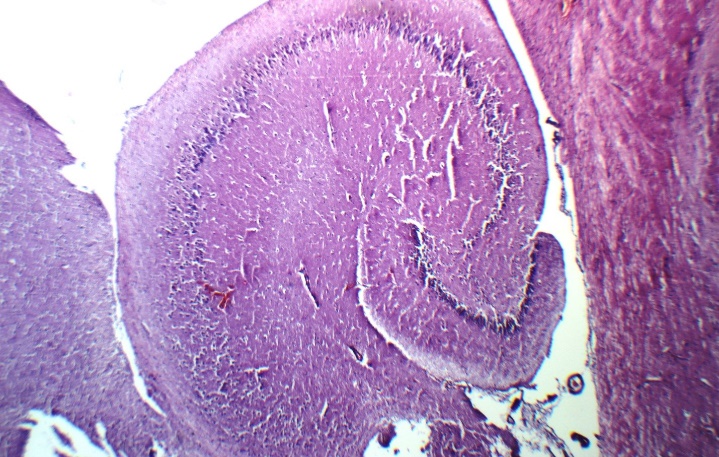

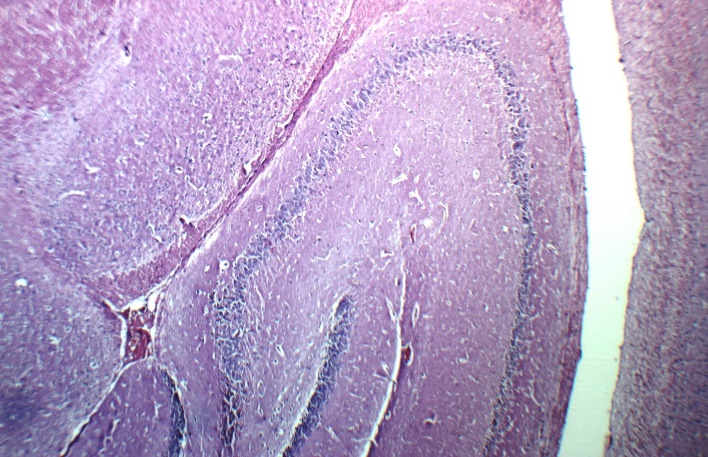


**D**

**C**


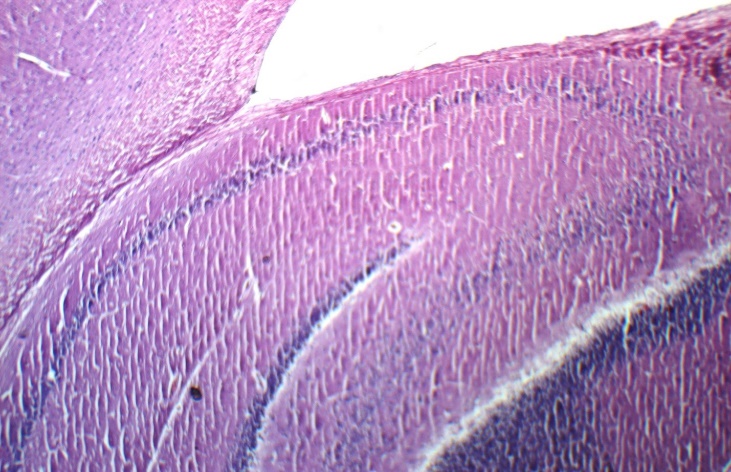

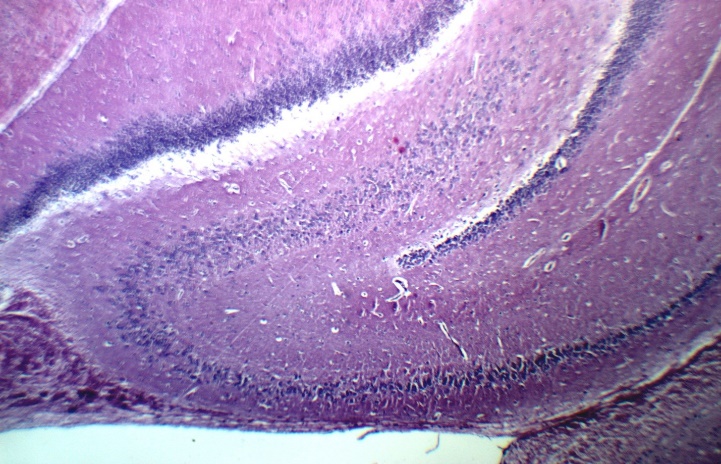


**F**

**E**


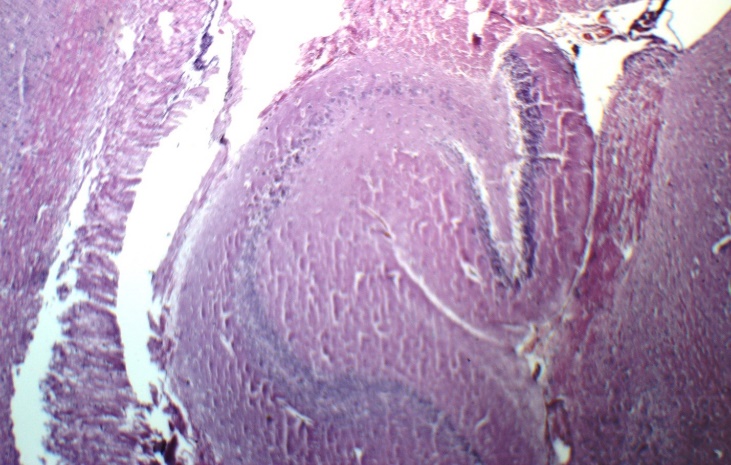


**G**

**Fig. 11: Histopathological investigation of brain tissue at different magnifications (hippocamus). (A)Negative control:** hippocampus showing average Cornu Amonis (CA1), (CA2), (CA3), average dentate gyrus (DG), and average blood vessels (black arrow) (H&E X 100) . **(B) Positive control:** hippocampus showing average Cornu Amonis (CA1), (CA2), (CA3), and average blood vessels (black arrow) (H&E X 100). **(C) SP 1:** hippocampus showing average Cornu Amonis (CA1), (CA2), (CA3), and average blood vessels (black arrow) (H&E X 100). **(D) SPN 1:** hippocampus showing average Cornu Amonis (CA1), (CA2), (CA3), and average blood vessels (black arrow) (H&E X 100). **(E) ECH 1:** hippocampus showing average Cornu Amonis (CA1), (CA2), (CA3), and average blood vessels (black arrow) (H&E X 100). **(F) ECH + SP:** hippocampus showing average Cornu Amonis (CA1), (CA2), (CA3), and average blood vessels (black arrow) (H&E X 100). **(G)** **ECH + SPN:** hippocampus showing average Cornu Amonis (CA1), (CA2), (CA3), and average blood vessels (black arrow) (H&E X 100).
